# Endocytosis of Wnt ligands from surrounding epithelial cells positions microtubule nucleation sites at dendrite branch points

**DOI:** 10.1371/journal.pbio.3002973

**Published:** 2025-01-06

**Authors:** Pankajam Thyagarajan, Hannah S. Mirshahi, Gregory O. Kothe, Nitish Kumar, Melissa Long, Bowofoluwa S. Abimbola, Alexis T. Weiner, Melissa M. Rolls

**Affiliations:** Biochemistry and Molecular Biology and the Huck Institutes of the Life Sciences, The Pennsylvania State University, University Park, Pennsylvania, United States of America; MRC Laboratory of Molecular Biology, UNITED KINGDOM OF GREAT BRITAIN AND NORTHERN IRELAND

## Abstract

Microtubule nucleation is important for microtubule organization in dendrites and for neuronal injury responses. The core nucleation protein, γTubulin (γTub), is localized to dendrite branch points in *Drosophila* sensory neurons by Wnt receptors and scaffolding proteins on endosomes. However, whether Wnt ligands are important is unknown. We found that Wnt secretion from epithelial cells was required for γTub localization to dendrite branch points. Using RNAi and mutant approaches, we demonstrated that Wnt4 and wntD both position γTub. Moreover, injury-induced increases in neuronal microtubule dynamics required Wnt secretion from epithelial cells. Overexpression of Wnts in epithelial cells increased microtubule dynamics to the same extent as axon injury indicating surrounding cells have an instructive role in neuronal nucleation. To determine how Wnt ligands concentrate microtubule nucleation at dendrite branch points, we tested whether endocytosis is restricted to specific regions of dendrites. Markers of clathrin-mediated endocytosis localized to puncta at branch points. Behavior of these puncta was sensitive to inhibition of endocytosis suggesting they represented endocytic sites. In addition to previously described colocalization of Wnt receptors and scaffolds with Rab5 endosomes, we identified a separate set of Wnt signaling puncta that colocalized with clathrin in dendrites. Moreover, γTub and Wnt scaffolding protein recruitment to branch points was reduced by clathrin RNAi, and injury-induced up-regulation of microtubule dynamics was sensitive to clathrin reduction. We propose that the localization of Wnt endocytic sites to dendrite branch points results in the local generation of microtubule nucleating endosomes.

## Abstract 2

A previous study in PLOS Biology found that Wnt receptors are housed in early endosomes and are required for dendritic microtubule nucleation. This Update Article shows that microtubule dynamics in dendrites can be influenced non-autonomously by surrounding epithelial cells.

## Introduction

Microtubule organization is critical for long term neuronal function. Changes in microtubule stability and microtubule associated proteins have been implicated in neurodegenerative diseases [1,2]. Increased microtubule dynamics occurs in response to axon injury or stress in *Drosophila* [3] and mammalian neurons [4], and is an important contributor to neuroprotection in *Drosophila* [3]. In healthy neurons, microtubules direct cargoes made in the cell body to axons and dendrites. Axons and dendrites have differently polarized microtubules with microtubule plus ends growing away from the cell body in axons and dendrites having either mixed or minus-end-out polarity [5–7]. This difference in polarity helps direct cargoes specifically to axons or dendrites [8,9].

An important contributor to neuronal microtubule dynamics and polarity is local microtubule nucleation mediated by the γTubulin ring complex (γTuRC) in axons and dendrites [10,11]. Reduction of nucleation alters microtubule dynamics and/or generates mixed microtubule polarity [12–15]. Moreover, disruption of microtubule nucleation impairs neuronal stability and dendrite growth and regeneration [3,15–19]. γTuRC-dependent nucleation is increased in dendrites by axon injury [3] and at axonal presynaptic sites by neuronal activity [20] suggesting that not only is local nucleation important for long-term neuronal resilience, but that its levels are regulated in response to specific neuronal states. At baseline, nucleation in dendrites is held in check by kinetochore proteins [18].

Although it has been clear for over a decade that microtubules are not nucleated at the centrosome in mature neurons [21,22], it has proven challenging to pinpoint sites and regulators of neuronal microtubule nucleation. The identification of endosomes as nucleation sites in tips of developing *Caenorhabditis elegans* dendrites [23] and at branch points of mature *Drosophila* dendrites [24] was a surprising development in the field. There are hints that additional nucleation sites remain to be characterized in dendrites. For example, in a developing *Drosophila* neuron hotspots of microtubule dynamics were observed near dendrite tips [23] and in mature *Drosophila* neurons endogenous γTubulin is seen in wider regions of terminal dendrites [25], and the nucleation activator centrosomin is seen in spots in dendrite tips [26]. However, our focus here is on the previously characterized nucleation sites at dendrite branch points of *Drosophila* Class I dendritic arborization neurons. These were initially defined as nucleation sites by localization of overexpressed and endogenous γTubulin [14], by concentration of nucleation activator centrosomin [26] and as sites of new microtubule emergence [14].

By using γTubulin-GFP localization at branch points as a screening tool, we identified Wnt signaling proteins as regulators of γTubulin concentration and showed they localize to endosomes at branch points in these Class I neurons where they act as sites of new microtubule initiation [24]. Moreover, when a Wnt receptor is reduced in larger Class IV neurons, dendrite regeneration is impaired and nucleation sites fail to be positioned in regrowing dendrites [17]. Wnt signaling had previously been shown to regulate the direction of microtubule growth in developing axons through the microtubule plus end-binding protein adenomatous polyposis coli (APC) [27]. The only hint that any Wnt signaling proteins might be involved in regulation at the microtubule minus end was the finding that the scaffolding protein Axin localizes to the centrosome in mammalian cell lines and helps recruit γTubulin [28]. In *Drosophila* dendrites, Axin seems to be the output of the pathway, and artificially relocalizing Axin to mitochondria is sufficient to recruit γTubulin to this site [24].

While Axin has been linked to positioning of nucleation sites in mammalian cultured cells [28] and *Drosophila* neurons [24], only in *Drosophila* have upstream regulators been identified. Proteins that position Axin and nucleation sites in dendrites include the scaffolding protein disheveled (dsh), kinases shaggy (sgg, GSK3b) and casein kinase 1 γ, receptors frizzled (fz) and frizzled 2 (fz2), and co-receptors arrow (arr, LRP5/6) and Ror [17,24]. These proteins (except perhaps Ror) all participate in canonical Wnt signaling [29–31], but the outputs of canonical Wnt signaling including APC and β-catenin are not required for dendritic nucleation, nor are Wnt signaling proteins specific to planar cell polarity (PCP) [24]. Because the dendritic pathway uses some, but not all, canonical Wnt signaling proteins we refer to it as apocryphal Wnt signaling [24].

Canonical Wnt signaling is activated by Wnt ligands binding to frizzled receptors in conjunction with the arrow (LRP5/6) co-receptor [32,33]. The PCP pathway also uses frizzleds, but Wnt ligands do not seem to play a central role in this context [34–36]. It is not known whether apocryphal Wnt signaling requires Wnt ligands like canonical Wnt signaling or acts without an absolute requirement for them like PCP.

Wnt ligands are large, secreted lipid-modified proteins that rely on their lipid group to interact with frizzleds. After crossing the endoplasmic reticulum membrane during synthesis, Wnts are glycosylated and subsequently have palmitoleic acid added by the porcupine (por) enzyme. Once lipidated, Wnts can be recognized by the dedicated chaperone evi/wntless (wls). Wls is required to transport Wnts through the secretory pathway and helps shield the lipid group [37,38]. Por and wls are thus essential players in Wnt signaling pathways that rely on Wnt ligands.

Although microtubule nucleation has not previously been suggested to be controlled by surrounding cells, the involvement of frizzleds, arrow and Ror in dendritic branch point nucleation [17,24] suggested the possibility of non-cell autonomous control. To determine whether neighboring cells might influence microtubule nucleation in *Drosophila* sensory dendrites, we used wls knockdown to reduce Wnt secretion from glia and epithelial cells. Glia wrap the cell body, axons, and proximal dendrites of *Drosophila* multidendritic sensory neurons [39], while epithelial cells contact, support and partially wrap more distal dendrites [40–42]. Reduction of wls in skin, but not glial, cells reduced γTubulin localization at dendrite branch points. We performed a candidate screen to identify skin cell-required Wnts and found Wnt4 and wntD act upstream of γTubulin localization. Localization of endogenous wls and Wnt4 suggests that Wnts are broadly secreted from skin cells. This raised the question of how widely distributed Wnts preferentially localize endosomal nucleation sites to branch points within dendrites. To address this question, we examined the site of endocytosis in dendrites. We found that rather than being randomly scattered over the cell surface, they were concentrated at dendrite branch points. We therefore hypothesized that apocryphal Wnt signaling is initiated at endocytic sites at branch points and this controls nucleation site position. Indeed, we observed a subset of dsh and Axin spots colocalizing with endocytic markers at branch points, and disruption of endocytosis reduced branch point localization of dsh and reduced the ability of neurons to up-regulate nucleation in response to axon injury. We propose that Wnt ligands from skin cells promote microtubule nucleation at branch points in neighboring dendrites and that the position of nucleation sites at branch points is determined by the site at which Wnts are endocytosed.

## Results

### Wnt secretion from epithelial cells is required for γTubulin localization at dendrite branch points

To determine whether surrounding cells influence microtubule nucleation at dendrite branch points, we used the *Drosophila* sensory neuron ddaE as a model system. Although *Drosophila* sensory dendrites are not postsynaptic, they share cellular features with dendrites of well-characterized vertebrate interneurons including presence of ribosomes [43], Golgi outposts [44], and minus-end-out microtubules [45]. Microtubule organization has been well characterized in ddaE [46]. Dendrites have mostly minus-end-out microtubules and nucleation sites are positioned at branch points [14], where they are important for maintaining microtubule polarity [14]. Local nucleation in ddaE dendrites is up-regulated in response to axon injury [3,24] and contributes to injury-induced stabilization [3]. ddaE neurons were previously used as a platform to identify regulators of microtubule nucleation at dendrite branch points. Wnt signaling proteins including arrow, fz, fz2, dsh, and Axin are required to concentrate γTubulin-GFP at branch points, to maintain microtubule polarity and to mediate the increase in microtubule dynamics elicited by axon injury [24]. We therefore used ddaE neurons to investigate the requirement for Wnt ligands in local microtubule nucleation at dendrite branch points.

**Fig 1:**
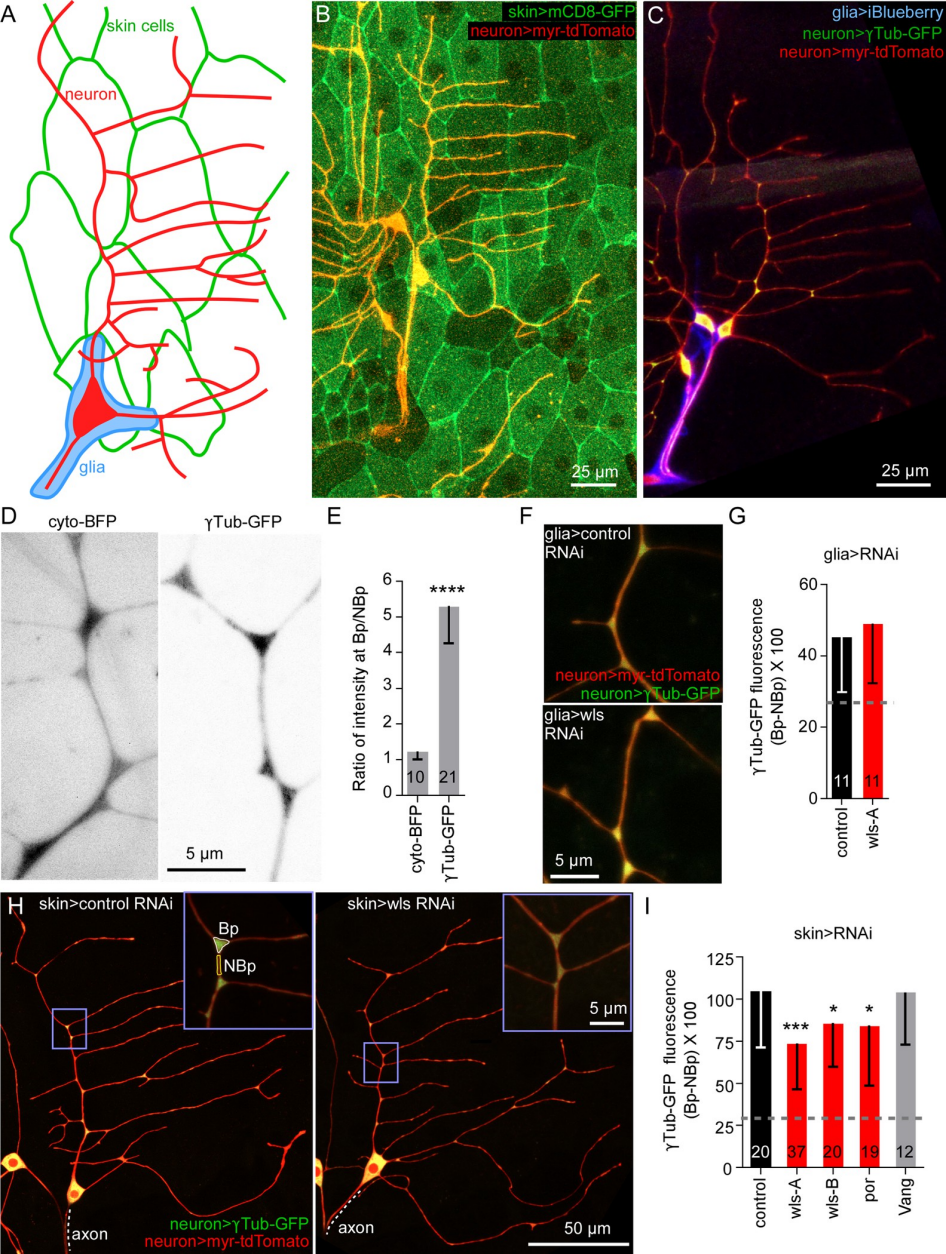
Wnt secretion from epithelial cells is required for γTubulin localization at dendrite branch points. (A) Diagram of the *Drosophila* Class I dorsal dendritic arborization (ddaE) neuron (red) with surrounding cells including skin cells (green) and glial cells (blue). (B) Overview of Class I neurons expressing QUAS-myr-tdTomato (red membrane marker) with the TMC-QF driver (Class 1 neuronal driver). ddaE is at the right and its sister ddaD is visible, but with part of dendrite arbor cut off, at left. Skin cells are labeled with membrane marker UAS-mCD8-GFP driven with A58-Gal4. (C) Overview of a ddaE neuron expressing QUAS-γTub-GFP and QUAS-myr-tdTomato with the TMC-QF driver. Glial cells are labeled with UAS-iBlueberry driven by repo-Gal4. (D) Representative images of cyto-BFP and γTub-GFP in the ddaE dorsal dendrite showing enriched fluorescence at Bps for γTub-GFP. (E) Quantitation of Bp enrichment of γTub-GFP and cyto-BFP. The numbers on the graphs represent the number of neurons analyzed in each category. ****, *P* < 0.0001 with Mann–Whitney test. (F) Representative images of neurons expressing QUAS-γtub-GFP and QUAS-myr-tdTomato with TMC-QF driver while the glial cell driver (repo-Gal4) expresses UAS-control-RNAi or UAS-wls-RNAi. (G) Quantitation of average γTub-GFP fluorescence (Bp-NBp) using the glial cell tester line. No difference was observed between control and wls-RNAi. *P* > 0.05 with Mann–Whitney test. (H) Overviews of ddaE neuron expressing QUAS-γTub-GFP and QUAS-myr-tdTomato with the TMC-QF driver with the skin cell driver (A58-Gal4) driving UAS-control-RNAi or UAS-wls-RNAi, quantified in I. (I) Quantitation of average γTub-GFP fluorescence (Bp-NBp) using the skin cell tester line. The dotted line on the graph is the predicted Bp-NBp value for a cytosolic fluorescent protein without targeting signals. ***, *P*< = 0.001, *, *P*< = 0.05 with Mann–Whitney test. Error bars in all graphs show standard deviation. Underlying data for the graphs can be found in S2 Table.

Whether Wnt ligands diffuse over long distances is controversial [47] so we first considered cells in direct contact with ddaE as potential sources of Wnts (Fig 1A). As in our previous studies, we used γTubulin-GFP localization at dendrite branch points as an initial proxy for position of nucleation sites [14,24]. This γTubulin-GFP is functional [14], but is overexpressed. We showed that it has a similar localization pattern to endogenous γTubulin [14], and it proved useful as a screening tool to identify proteins that position nucleation sites in dendrites; all proteins identified as important for γTubulin-GFP localization were subsequently confirmed to influence microtubule polarity and/or dynamics in response to injury in dendrites [24]. To reduce Wnt secretion from epithelial and glial cells, we used the Gal4-UAS system [48] to express hairpin RNAs targeting wls. A58-Gal4 drives expression in most larval epithelial cells (Fig 1B) and Repo-Gal4 drives expression in glial cells that wrap ddaE axons (Fig 1C). To visualize γTubulin-GFP in Class I dendritic arborization neurons including ddaE, we used a second binary expression system, QF-QUAS [49]. By pairing 2 binary expression systems, we could perform cell type-specific RNAi in candidate neighboring cells using Gal4-UAS while visualizing reporters in neurons with QF-QUAS. QUAS-γTubulin-GFP was enriched at branch points compared to cytoplasmic BFP (Fig 1D and 1E) similar to UAS-γTubulin-GFP [14]. To compare -γTubulin-GFP enrichment at branch points across genotypes, we measured fluorescence intensity at branch points (Bp) and in between branch points (nBp). We then subtracted the nBp average from the Bp average as in previous work [24]. The fluorescent values are from 16-bit images, so are in the 1,000s range so we divided by 100 to simplify the y axis labels. Because even cytosolic fluorescent proteins are slightly enriched at branch points likely based on the larger volume of branch points compared to neighboring regions (Fig 1E and [14,24]), the predicted baseline of no enrichment (but larger branch point volume) was calculated and is indicated with a dashed line on the graph. We approximated this baseline using expression of cytosolic fluorescent proteins with the same driver. The enrichment of cytosolic proteins is generally 1.2- to 1.3-fold at branch points (Fig 1E and [24]) so we multiplied the mean NBp value of control data set(s) by this factor to estimate fluorescence of proteins with no localization signals at branch points. Expression of wls RNAi hairpins in glial cells had no effect on γTubulin-GFP localization to dendrite branch points (Fig 1F and 1G). In contrast, epithelial expression of 2 different wls RNAi hairpins reduced levels of γTubulin-GFP at branch points (Fig 1H and 1I). Similarly, por RNAi hairpin expression in epithelial cells also reduced branch point localization of γTubulin (Fig 1I). In the PCP pathway Van Gogh (Vang) protein on neighboring cells binds the frizzled extracellular domain [50] so we also expressed Vang RNAi hairpins in skin cells; γTubulin localization in neurons was unaffected (Fig 1I). We previously expressed wls RNAi hairpins in Class I neurons and saw no effect on γTubulin localization [24]. These data together suggest that skin cells are a source of Wnt ligands that recruit γTubulin to dendrite branch points.

### Skin cell-derived Wnt4 and wntD are required for γTubulin localization at dendrite branch points

**Fig 2:**
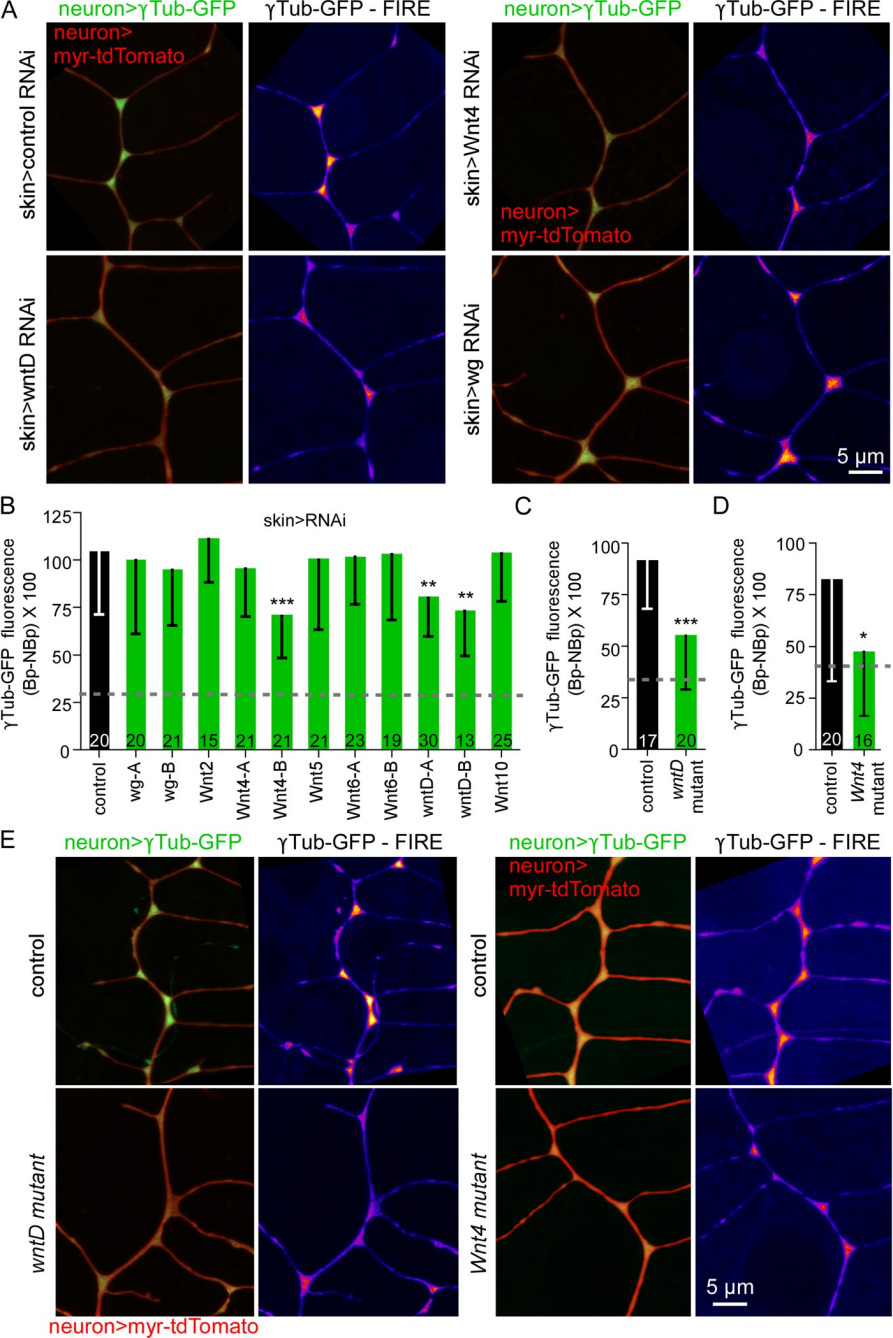
Skin cell-derived Wnt4 and wntD are required for γTubulin localization at dendrite branch points. (A) Representative images of a region of the ddaE dorsal comb dendrite expressing QUAS-myr-tdTomato and QUAS-γTub-GFP with TMC-QF, with A58-Gal4 driving UAS-RNAi hairpins in skin cells. (B) Quantitation of γTub-GFP fluorescence (Bp-NBp) in ddaE using the skin cell tester line crossed to different RNAi hairpin lines. ***, *P*< = 0.001, **, *P*< = 0.01 with Mann–Whitney test. (C, D) Quantitation of γTub-GFP fluorescence (Bp-NBp) using wntD mutant and Wnt4 mutant. ***, *P*< = 0.001, *, *P*< = 0.05. Numbers on bars are number of cells analyzed and error bars are standard deviation. Units are raw fluorescent values from 16 bit images. For the FIRE lookup table (LUT) values were set from 0 to 30,000 for all images. Numerical data used in the graphs can is in S2 Table. (E) Example images of γTub-GFP fluorescence in control and *Wnt* mutant animals.

After identifying the skin cells as a source of Wnt ligands that act upstream of branch point γTubulin localization, we wanted to identify the specific Wnt involved. There are 7 different Wnts in *Drosophila* [51] and we used the same double binary expression system described above to express RNAi hairpins targeting each Wnt in skin cells while assaying γTubulin localization at branch points. Where possible we used 2 different RNAi lines to target a specific Wnt as the effectiveness of RNAi lines varies. Knockdown of both Wnt4 and wntD in epithelial cells reduced γTubulin-GFP at branch points (Fig 2A and 2B). Both wntD RNAi lines had similar effects, but only one of the Wnt4 lines affected γTubulin localization, likely due to ineffective knockdown by the other line. There was no significant change in the γTubulin-GFP fluorescence in the region between branch points (S1 Fig) suggesting that concentration at branch points rather than overall levels of γTubulin-GFP was affected by reducing Wnt4 and wntD in skin cells. To confirm the role of Wnt4 and wntD, we assembled *Drosophila* lines that included mutant alleles with neuronally expressed γTubulin-GFP. The *wntD*^*KO2*^ allele is a null mutant that is homozygous viable [52]. In homozygous *wntD*^*KO2*^ larvae, we observed a significant decrease in intensity of γTubulin at branch points (Fig 2C and 2E), but not between branch points (S1 Fig). Most animals homozygous for null or strong loss of function *Wnt4* alleles including *Wnt4*^*C1*^ and *Wnt4*^*EMS23*^ die in early larval stages [53], but we were able to recover *Wnt4*^*C1*^/*Wnt4*^*EMS23*^ transheterozygous larvae for analysis. In this background, recruitment of γTubulin to branch points was also reduced (Fig 2D and 2F); this was the only condition where γTubulin-GFP between branch points was also reduced (S1 Fig), perhaps related to the overall health of the animals. These data together demonstrate that skin cell-derived Wnt4 and wntD ligands are required to concentrate γTubulin at dendrite branch points.

### Wnt4 and wntD from skin cells can regulate microtubule dynamics in dendrites

**Fig 3:**
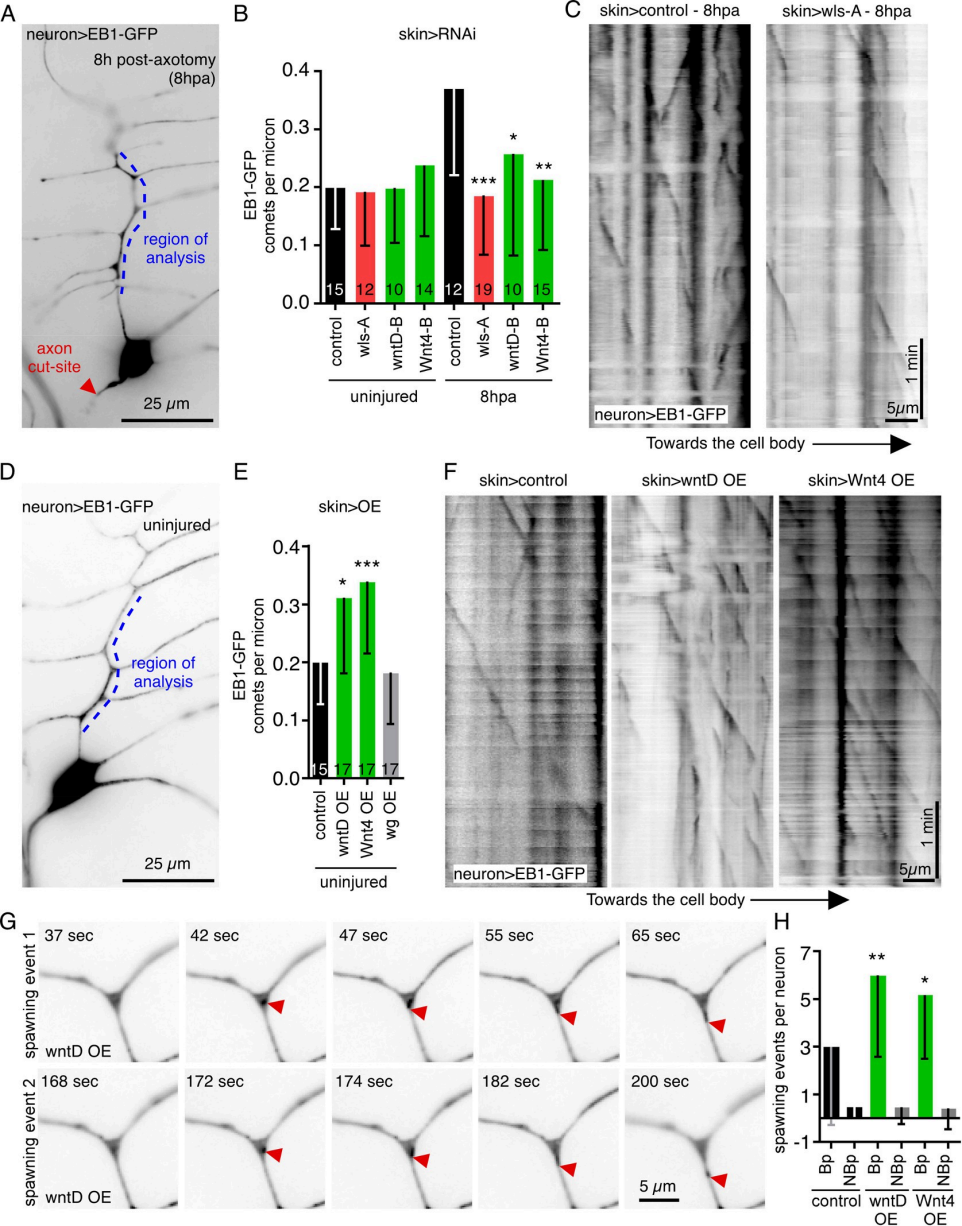
Wnt4 and wntD from skin cells regulate microtubule dynamics in dendrites. (A) Overview of ddaE neuron expressing QUAS-EB1-GFP with the TMC-QF driver. The red arrowhead points to the site of axon injury and the blue dotted line marks the region of interest for quantitating the number of EB1-GFP comets. (B) Quantitation of number EB1-GFP comets per micron in dendrites with UAS-RNAi hairpins expressed using the skin-cell driver (A58-Gal4). Comets were quantitated in uninjured neurons and in neurons 8 h post-axotomy (hpa). *, *P*< = 0.05, **, *P*< = 0.01, ***, *P*< = 0.001 with Mann–Whitney test. (C) Representative kymographs showing EB1-GFP comets 8 h post axotomy in control RNAi and wls RNAi neurons. (D) Overview of ddaE neuron expressing QUAS-EB1-GFP with the TMC-QF driver in uninjured neurons. The region of interest is marked in blue. (E) Quantitation of number of EB1-GFP comets per micron in dendrites while UAS-driven transgenes are overexpressed (OE) in skin cells using A58-Gal4. (F) Representative kymographs showing EB1-GFP comets when UAS-control-RNAi, UAS-wntD OE and UAS-Wnt4 OE are expressed in skin cells. *, *P*< = 0.05, ***, *P*< = 0.001 with Mann–Whitney test. (G) Examples of EB1-GFP comets “spawning” (initiating from a region free of EB1-GFP puncta in at least 3 preceding frames) are shown. (H) Spawning events from the data set in D–F were categorized based on whether they occurred at branch points (Bp) or not at branch points (NBp). A Mann–Whitney test was used for comparison and *n* = 17 neurons for each genotype and numerical data is in S2 Table.

While we have previously validated localization of γTubulin-GFP as a reporter of nucleation sites [14,24], we also wished to use a functional assay to determine whether Wnts from skin cells can modulate neuronal microtubule nucleation. We have shown that the number of growing microtubule plus ends in dendrites increases in response to axon injury [54] and that this increase depends on microtubule nucleation [3]. Microtubule severing could increase number of plus ends in response to injury, but this increase after axon injury is selectively sensitive to reduction of nucleation regulators including γTubulin, Plp, cnn, and γTuRC subunits [3,24] and is not affected by reduction of severing proteins [3]. It is therefore a useful assay for identification of regulators of dendritic microtubule nucleation. Moreover, this injury-dependent increase is sensitive to reduction of Wnt receptors and scaffolding proteins in neurons [24]. EB1 is a microtubule end-binding protein that recognizes growing ends and has been previously used to monitor microtubule polarity and dynamics in mammalian [55] and *Drosophila* neurons [56]. We therefore generated a QUAS-controlled EB1-GFP to monitor microtubule behavior in ddaE neurons while using UAS-RNAi expression in skin cells to reduce Wnt secretion. As expected, the number of growing plus ends was strongly up-regulated in control neurons 8 h after axons were removed using laser microsurgery (Fig 3A–3C). To test whether increased microtubule dynamics in response to axon injury is due to an increase in γTubulin protein levels, we examined an γTubulin tagged at the endogenous locus with sfGFP [25]. This tagged γTub-sfGFP was reliably detected in the ddaE soma, and occasionally visible above background at dendrite branch points (S2A and S2B Fig). No change in γTub-sfGFP was seen at 24 h after axon injury (S2C Fig), when microtubule dynamics remains highly up-regulated [3] suggesting that regulation of nucleation is modulated in response to axon injury. The injury-induced increase in microtubule dynamics was suppressed when Wnt secretion was blocked in skin cells by wls RNAi, or when Wnt4 or wntD were reduced in skin cells (Fig 3B and 3C). No difference in microtubule dynamics or polarity was seen in uninjured neurons (Figs 3B and S1D–S1F) consistent with previous results indicating that very strong reduction of neuronal microtubule nucleation is required to alter baseline microtubule behavior in these cells [14]. In summary, these results suggest that secretion of Wnt4 and wntD from skin cells is important for neurons to regulate microtubule nucleation in dendrites.

Epithelial-derived Wnts could influence dendritic microtubule nucleation at branch points in several ways. They could act as a permissive signal that allows microtubule nucleation sites to localize to branch points, but do not regulate nucleation activity once a threshold that supports localization is reached. Alternatively, levels of Wnt signaling could be instructive and could modulate levels of nucleation in branch points. To distinguish between permissive and instructive roles of epithelial Wnts, we overexpressed Wnt4 and wntD in skin cells using the Gal4-UAS system and assayed microtubule dynamics in neurons using EB1-GFP expressed with the QF-QUAS system. Expression of either Wnt in skin cells resulted in a significant increase in the number of growing microtubule plus ends in dendrites (Fig 3E and 3F and S1 Movie). Expression of a different Wnt, wg, that did not have an effect on γTubulin-GFP localization did not increase microtubule dynamics (Fig 3E). To determine whether this increase in microtubule dynamics was linked to nucleation at branch points, we analyzed microtubule “spawning.” We define spawning as the appearance of new growing plus ends [14]. Most new plus ends in the main trunk of the ddaE dorsal dendrite initiate at branch points (Fig 3G and 3H). When wntD or Wnt4 was overexpressed the number of spawning events at branch points, and not between branch points, increased, consistent with increased nucleation at branch points (Fig 3H). This data suggests that Wnt ligands from skin cells have an instructive role in modulating levels of microtubule nucleation at dendrite branch points.

### Markers of clathrin-mediated endocytosis localize to dendrite branch points

Having shown that Wnt ligands from surrounding cells act upstream of dendritic branch point microtubule nucleation, we wished to understand how Wnt signaling might localize nucleation sites to specific regions of dendrites. Rather than being scattered randomly or uniformly distributed in dendrites, nucleation sites are concentrated at dendrite branch points [14]. We therefore hypothesized that some aspect of Wnt signaling could control this regionalization. We considered several steps that could be spatially restricted to pattern nucleation sites in dendrites. First, Wnt secretion could be targeted to regions of epithelial cells abutting dendrite branch points. Second, Wnt receptors could be localized specifically to branch points. Third, endocytosis of Wnt signaling proteins could be restricted to branch points. We previously examined the localization of tagged fz in ddaE neurons and saw uniform plasma membrane distribution as well as localization to endosomes [24]. To determine whether Wnt secretion might be targeted to regions near branch points, we examined the localization of tagged wls and Wnt4. Wls[ExGFP] flies were engineered such that a version of wls with GFP inserted in the fourth loop on the ER-luminal side replaces the *wls* gene [36]. We used Cas9-mediated genome engineering to introduce the mNeonGreen (mNG [57]) coding sequence at the 3′ end of the *Wnt4* gene before the stop codon. Most (85% to 90%) *Wnt4* mutant animals die as larvae, with the surviving adults exhibiting sterility [53]. We were able to generate a homozygous Wnt4-mNG stock suggesting that the endogenous tagged Wnt4 is functional. Wls[ExGFP] and Wnt4-mNG were present in a similar reticular pattern throughout epithelial cells (S3A and S3B Fig). We did not see any evidence for enrichment of either protein in regions near ddaE dendrite branch points. Note that Wnt4-mNG expression was clearly observed in epithelial cells (S3A Fig) but may also be present in glia and muscle (S3C and S3D Fig). Because neither Wnt ligands nor receptors seemed to be targeted to dendrite branch points, we considered the last possibility: that endocytic sites might be localized specifically to branch points. Wnt signaling can initiate at plasma membrane sites called signalosomes, but in many contexts is enhanced by clathrin-mediated endocytosis of ligand-receptor complexes [32,33,58].

**Fig 4:**
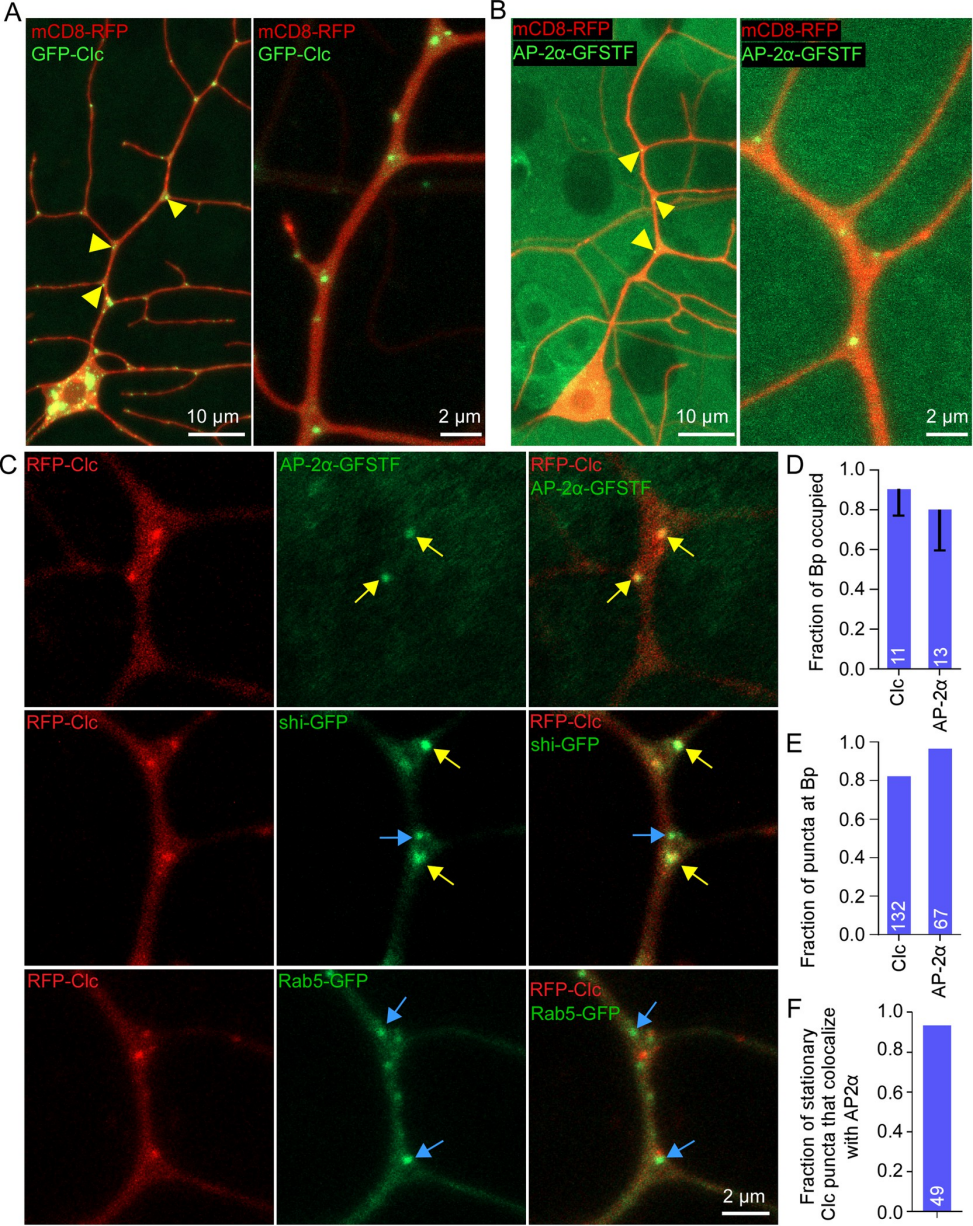
Markers of clathrin-mediated endocytosis localize to dendrite branch points. (A) Overview (left) and zoomed-in image (right) of ddaE neurons expressing UAS-mCD8-RFP (red membrane marker) and UAS-GFP-Clc (clathin light chain) with the Class I neuron-specific driver 221-Gal4. Yellow arrowheads mark GFP-Clc puncta localized to dendrite branch points. (B) Overview (left) and zoomed-in image (right) of endogenous AP-2α-GFSTF protein-trap (Adaptor Protein-2α) and ddaE neurons labeled with UAS-mCD8-RFP (red membrane marker) using 221-Gal4. Yellow arrowheads mark endogenous AP-2α-GFSTF puncta localized to dendrite branch points. (C) First row: Representative image showing localization of endogenous AP-2α-GFSTF puncta with UAS-RFP-Clc puncta expressed in ddaE dendrites with TMC-Gal4 driver. Second row: Representative image showing localization of UAS-shi-GFP (shibire/dynamin) with UAS-RFP-Clc expressed in ddaE dendrites with TMC-Gal4 driver. Third row: Representative image showing localization of UAS-Rab5-GFP puncta with UAS-RFP-Clc expressed in ddaE dendrites. Yellow arrows indicate puncta that colocalize with RFP-Clc while blue arrows indicate puncta that do not colocalize with RFP-Clc. (D) Quantitation of number of branch points occupied with RFP-Clc or AP-2α-GFSTF puncta divided by total number of branch points in focus for each ddaE dendrite. The numbers on the bar graph represent the number of neurons analyzed. (E) Quantitation of total number of puncta at branch point regions compared to non-branch point regions in ddaE dendrites. The numbers on the bar graph represent the total number of puncta counted. (F) Quantitation of number of stationary RFP-Clc puncta that colocalize with AP-2α-GFSTF; the number is total puncta counted and values can be found in S2 Table.

Tagged clathrin light chain (Clc) has been used to monitor clathrin-mediated endocytosis in many systems including *Drosophila* [59]. Indeed, a previous report noted that tagged Clc was frequently observed in branch points in Class IV dendritic arborization neurons, the most complex type of larval sensory neurons in *Drosophila* [60]. We therefore examined the localization of tagged Clc as well as two other endocytic proteins in ddaE neurons. Clear GFP-Clc puncta were observed in about 90% of branch points along the trunk of the main dorsal ddaE dendrite (Fig 4A and 4D). To confirm that these spots were not induced by overexpression of Clc using the Gal4-UAS system, we visualized tagged endogenous AP-2α (AP-2α-GFSTF) [61]. AP-2α is a subunit of an adaptor complex that links cargo to the clathrin coat. Like GFP-Clc, AP-2α-GFSTF formed punctate structures at about 80% of branch points (Fig 4B and 4D). Not only were most branch points occupied by tagged Clc and AP-2α, but over 80% of puncta formed by either marker localized to branch points in the central region (main trunk and base of side branches) of the dendrites imaged (Fig 4E). We also examined a third marker of endocytosis, dynamin (called shibire, or shi, in flies). Dynamin localizes to the neck of clathrin coated pits and helps them pinch from the plasma membrane. UAS-shi-GFP [62] also formed puncta at branch points, although these were more variable than puncta formed by the other endocytic markers (Fig 4C). Colocalization between many AP-2α-GFSTF and RFP-Clc and shi-GFP and RFP-Clc puncta (Fig 4C and 4F and S2 Movie) suggested that the puncta are biologically meaningful. In contrast, although Clc and Rab5 both form puncta at branch points (in the main trunk of the ddaE dendrite 63% of Rab5 puncta were at branch points), these are distinct and do not overlap (blue arrows lower row Fig 4C). In sum, localization of endocytic markers suggested that endocytic sites could be targeted to dendrite branch points.

### Dendrite branch points are sites of clathrin-mediated endocytosis

While the presence of endocytic proteins at dendrite branch points is consistent with these being sites of endocytosis, clathrin can also localize to transport packets that appear as puncta in neurons [63]. To begin to characterize the clathrin puncta at branch points, we performed time-lapse imaging of GFP-Clc in dendrites over several minutes. We observed 2 types of puncta in branch points: large stationary puncta and highly dynamic, and often dimmer, puncta that swarmed at branch points (S3 Movie). To further characterize the time scale of stable puncta residence, we implemented live imaging of the whole larva for longer time periods using the LarvaSPA method [64]. Surprisingly, individual puncta could remain at branch points for the entire 90 min of imaging (S4 Movie). We also saw that stationary patches could exchange GFP-Clc with the moving population, with new Clc puncta separating from the stationary puncta or moving Clc puncta merging with the stationary patch (S4 Movie and S4 Fig).

**Fig 5:**
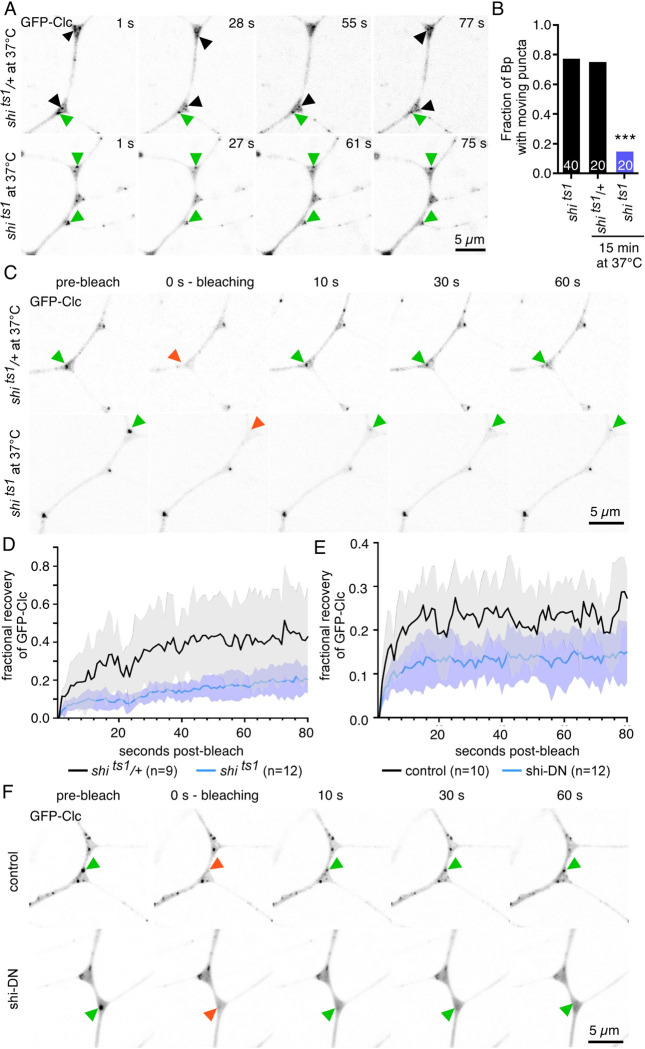
Dendrite branch points are sites of clathrin-mediated endocytosis. (A) Representative images from movies of GFP-Clc dynamics in control (*shi*^*ts1*^ /+) and *shi*^*ts1*^ animals after 15 min at 37C. Green arrowheads point to stationary puncta while black arrowheads point to moving puncta. Moving puncta are rare in the *shi*^*ts1*^ animals. (B) Quantitation of the presence of moving puncta divided by the total number of branch points for each condition. The left bar shows moving puncta in *shi*^*ts1*^ animals at room temperature. (C) Representative images from FRAP experiments in which a stationary GFP-Clc puncta (green arrowhead) was bleached (red arrowhead) and recovery was monitored with time lapse imaging. The first row shows control (*shi*^*ts1*^ /+) animals while the second row shows the *shi*^*ts1*^ animals. (D) Quantitation of fractional recovery of GFP-Clc intensity from the FRAP assay performed in control (*shi*^*ts1*^ /+) and *shi*^*ts1*^ animals. (E) Quantitation of fractional recovery of GFP-Clc intensity from the FRAP assay performed in control and shi-DN animals; data values are in S2 Table. (F) Representative images from the FRAP assay in control and shi-DN animals.

Based on the behavior of GFP-Clc, we hypothesized that the stable puncta may be endocytic patches or plaques [65] with the dynamic spots representing transport intermediates or endocytosed vesicles. If either type of puncta was associated with endocytosis rather than long-distance transport, blocking endocytosis should affect its dynamics. We used the classic temperature-sensitive allele of dynamin (*shi*^*ts1*^) to arrest endocytosis just before scission [66]. When animals containing only this *shi* allele are raised at the permissive temperature presynaptic terminals are normal, but at the non-permissive temperature at which flies become paralyzed light bulb-shaped plasma membrane invaginations with rings around their necks accumulate and this can be reversed by returning animals to permissive temperature [66]. We leveraged the position of the *shi* gene on the X chromosome to generate hemizygous male progeny with only the *shi*^*ts1*^ allele (*shi*^*ts1*^/Y). Mobile and stationary GFP-Clc puncta were observed in these males at the permissive temperature and in heterozygous females at the non-permissive temperature (Fig 5A and 5B and S5 Movie). In contrast, almost no moving puncta were observed after 15 min at 37C in *shi*^*ts1*^ animals while stationary puncta remained (Fig 5A and 5B and S5 Movie). This result tied the moving puncta to endocytosis but left the relationship of stationary ones ambiguous. It has previously been shown that GFP-Clc exchanges in plasma membrane patches in HeLa cells and that this exchange is partially reduced when endocytosis is inhibited by a dominant-negative dynamin or reduction of auxilins [67,68]. We therefore used fluorescence recovery after photobleaching (FRAP) to monitor GFP-Clc exchange in stationary puncta. When we bleached the non-motile GFP-Clc spots in heterozygous (control) animals at the restrictive temperature (Fig 5C and 5D and S6 Movie), we observed partial recovery of fluorescence indicating some exchange of Clc between membrane and cytosolic pools. In contrast, in the larvae with only the *shi*^*ts1*^ allele at restrictive temperature, we observed very little recovery (Fig 5C and 5D and S6 Movie). We also expressed a dominant-negative dynamin (shi-DN) constitutively to reduce endocytosis over a longer time scale at normal temperature. In this background, large stationary GFP-Clc puncta were present at dendrite branch points (Fig 5F) and very little recovery was seen after photobleaching (Fig 5E and 5F and S7 Movie). The genotype and temperature-matched control for this experiment also recovered less than that for the *shi*^*ts1*^ experiment (Fig 5D and 5E), perhaps because of the temperature difference (room temperature compared to 37C). We conclude that the behavior of both motile and stationary GFP-Clc puncta is sensitive to reduction of endocytosis, indicating that both types of labeled structures are likely to be sites or products of endocytosis. The fact that the larger stationary structures remain in the presence of temperature-sensitive and dominant-negative dynamin suggests that these are clathrin patches localized to the plasma membrane.

### Wnt signaling proteins form puncta that colocalize with endosomes or clathrin

**Fig 6:**
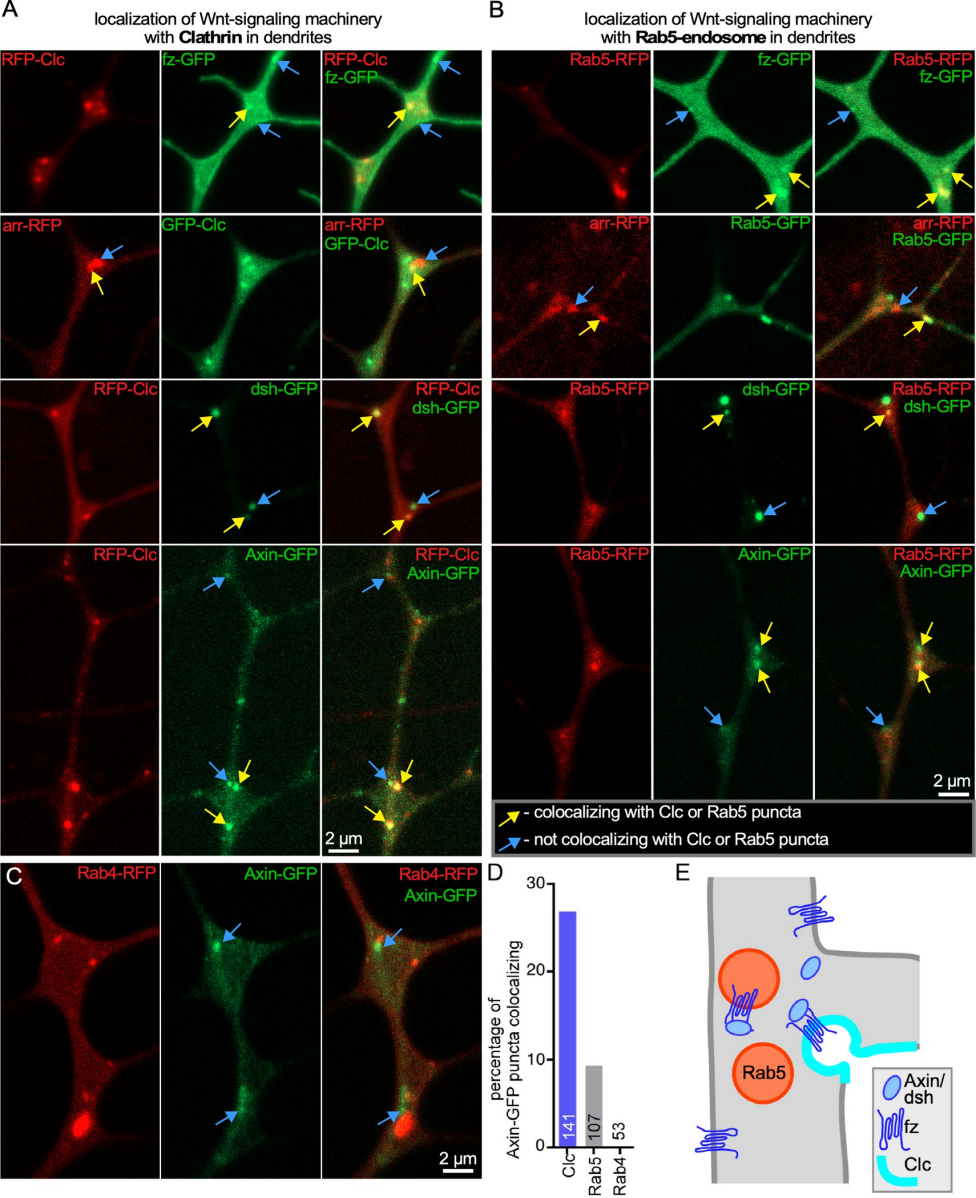
Wnt signaling proteins form puncta that colocalize with endosomes or clathrin. (A) Example images show UAS-driven Wnt signaling proteins expressed with UAS-RFP or GFP-Clc. Two subsets of puncta were observed: puncta that colocalized with stationary Clc (yellow arrows) and puncta that do not colocalize with stationary Clc (blue arrows). (B) Example images of UAS-driven Wnt signaling proteins with UAS-Rab5-RFP or GFP are shown. Two subsets of puncta were observed: those that colocalize with Rab5 (yellow arrows) and those that do not colocalize (blue arrows). (C) Representative images of UAS-Axin-GFP with UAS-Rab4-RFP are shown. No colocalizing puncta were observed. (D) Quantitation of total number of Axin-GFP puncta and the percentage that colocalize with RFP-Clc or RFP-Rab5 or RFP-Rab4; values are in S2 Table. (E) Schematic diagram showing that Wnt signaling proteins can colocalize with both stationary clathrin puncta and Rab5 endosomes. There are also populations of Wnt proteins that do not colocalize with either Clc or Rab5, and Rab5 endosomes that do not colocalize with Wnt proteins.

Having shown that clathrin-mediated endocytosis occurs at dendrite branch points, we wished to determine the relationship between endocytosis and local microtubule nucleation in dendrites. We previously observed the Wnt receptor frizzled (fz) and co-receptor arrow (arr) as well as scaffolding proteins Axin and disheveled (dsh) in puncta at branch points. Some of these puncta colocalized with Rab5, a marker of early endosomes [24]. We expressed tagged fz, arr, Axin, and dsh with tagged Rab5 or with Rab4 as a negative control. We saw clear, although infrequent, examples of each of these markers colocalizing with Rab5 (Fig 6B), and in some cases the marker moved together with Rab5 confirming that they were in the same structure (S8 Movie). In contrast, we never observed Wnt signaling proteins in puncta marked with Rab4 (Fig 6C and 6D). Because only a subset of Wnt protein puncta colocalized with Rab5, we considered that some of the remaining puncta might be associated with clathrin patches; indeed nematode and vertebrate Disheveled binds directly to AP-2 [69]. We therefore expressed the same set of Wnt signaling proteins with tagged Clc. As with Rab5, a subset of the puncta colocalized with Clc. For Axin, we quantified how many puncta overlapped with each marker. Counting any cluster visible in the image area (main trunk of ddaE dorsal dendrite and base of side branches), we found about 25% colocalized with Clc, 8% to 10% colocalized with Rab5, but none with Rab4 (Fig 6D). The remaining Axin puncta that did not colocalize with either compartment could overlap a different type of endosome not labeled with Rab5 or could represent clusters of Axin that form ectopically due to overexpression. Similarly, only a subset of stationary Clc puncta (26%) or Rab5 puncta (23%) were found at sites with Axin-GFP. Nevertheless, the colocalization of some Wnt signaling puncta with tagged Clc suggested a relationship with local endocytosis at branch points (Fig 6E).

### Localization of Wnt signaling scaffold dsh at dendrite branch points requires clathrin

**Fig 7:**
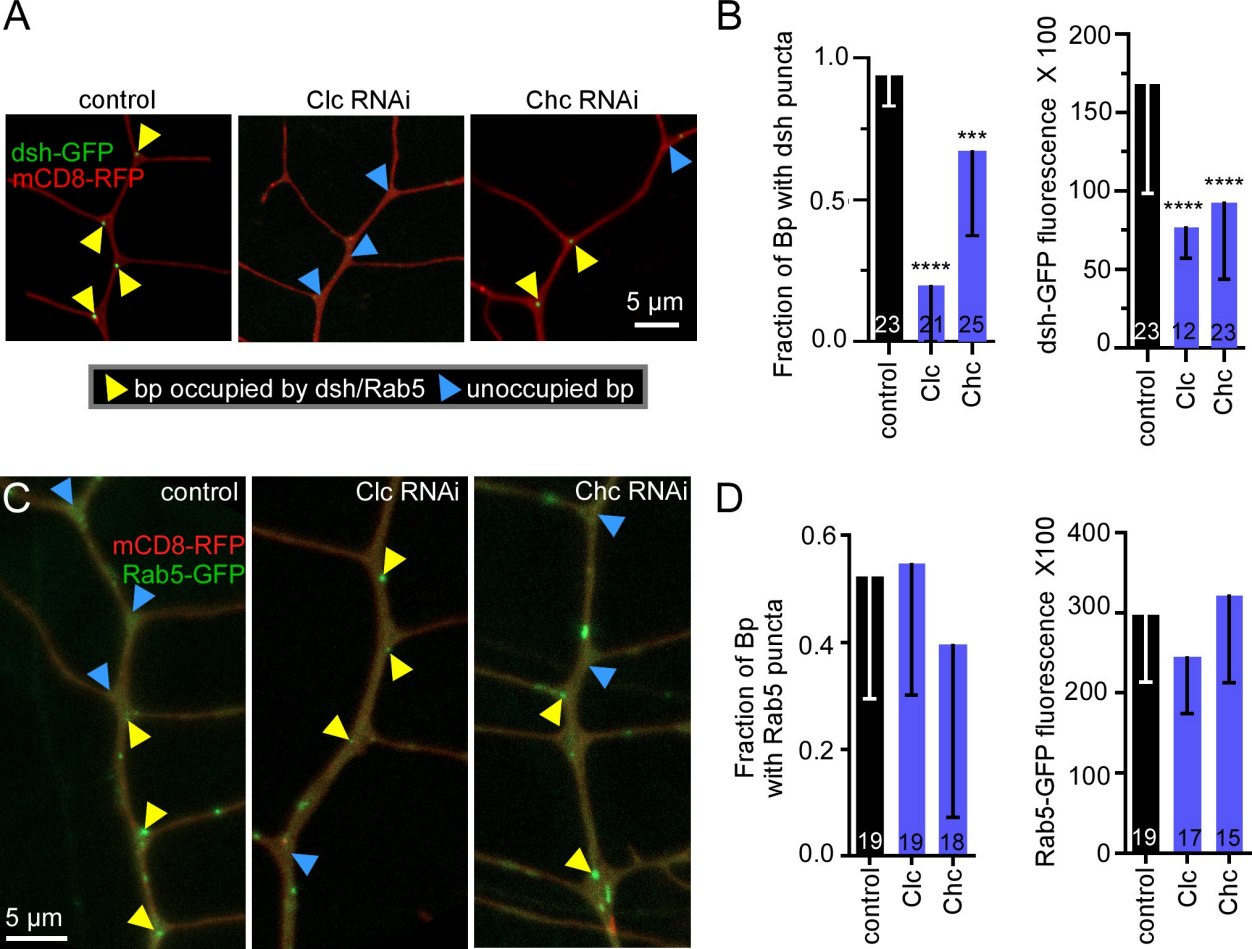
Localization of Wnt signaling proteins in dendrites requires clathrin. (A) Representative images of UAS-dsh-GFP localization in UAS-control-RNAi and UAS-clathrin-RNAi with 221-Gal4 driving the expression of UAS-dsh-GFP and UAS-mCD8-RFP (red membrane marker). The yellow arrowheads show branch points occupied with dsh-GFP puncta while the blue arrowheads show branch points not occupied with dsh-GFP puncta. (B) Quantitation of occupancy of branch points with dsh-GFP puncta in different conditions and the mean fluorescence intensity of the dsh-GFP puncta. *, *P*< = 0.05, ***, *P*< = 0.001, and ****, *P*< = 0.0001 with Mann–Whitney test. (C) Representative images of UAS-Rab5-GFP localization in UAS-control-RNAi and UAS-clathrin-RNAi conditions with 221-Gal4 driving expression. The yellow arrowheads show branch points occupied with Rab5-GFP puncta while the blue arrowheads show branch points not occupied with Rab5-GFP puncta. (D) Quantitation of occupancy of Rab5-GFP puncta in different conditions and the mean fluorescence intensity of the Rab5-GFP puncta. N’s on graphs are number of neurons analyzed and error bars are standard deviation; underlying data can be found in in S2 Table.

To further investigate the relationship between endocytosis and the apocryphal Wnt pathway, we used RNAi to disrupt endocytosis and assayed the effect on dsh and Rab5. Targeting clathrin by siRNA in mammalian cells strongly reduces endocytosis [68]. Both Clc and clathrin heavy chain (Chc) RNAi reduced intensity of RFP-Clc puncta at branch points; Clc more strongly than Chc RNAi (S5 Fig). However, in both cases RFP-Clc puncta were still detectable at many branch points (S5 Fig) suggesting partial reduction of endocytosis. Clc and Chc RNAi did not alter Rab5 puncta number or intensity in dendrites (Fig 7C and 7D), perhaps because Rab5 endosomes can be transported into dendrites by dynein [70] as well as being generated locally. A small subset being generated locally would be consistent with the 23% overlap of Rab5 puncta with Axin mentioned above. Dsh forms very discrete puncta at branch points, and acts upstream of Axin in apocryphal Wnt signaling [24]. We therefore used this marker to test the effect of reduction of clathrin on Wnt signaling. Clc and Chc RNAi reduced occupancy of branch points by of dsh-GFP puncta and remaining puncta were dim (Fig 7A and 7B). While overall cell shape appeared normal in these conditions, and no change in branch point number was observed (S6A and S6B Fig), Clc RNAi also reduced dsh-GFP fluorescence in the cell body (S6C Fig). Chc RNAi had a weaker effect on number of dsh-GFP occupied branch points (Fig 7B) consistent with its weaker effect on RFP-Clc (S5 Fig). This data suggests that concentration of Wnt signaling proteins at branch points is sensitive to reduction of endocytosis, and this effect scales with strength of reduction of endocytic patches in dendrites.

### Microtubule nucleation at dendrite branch points can be regulated by clathrin-mediated endocytosis

**Fig 8:**
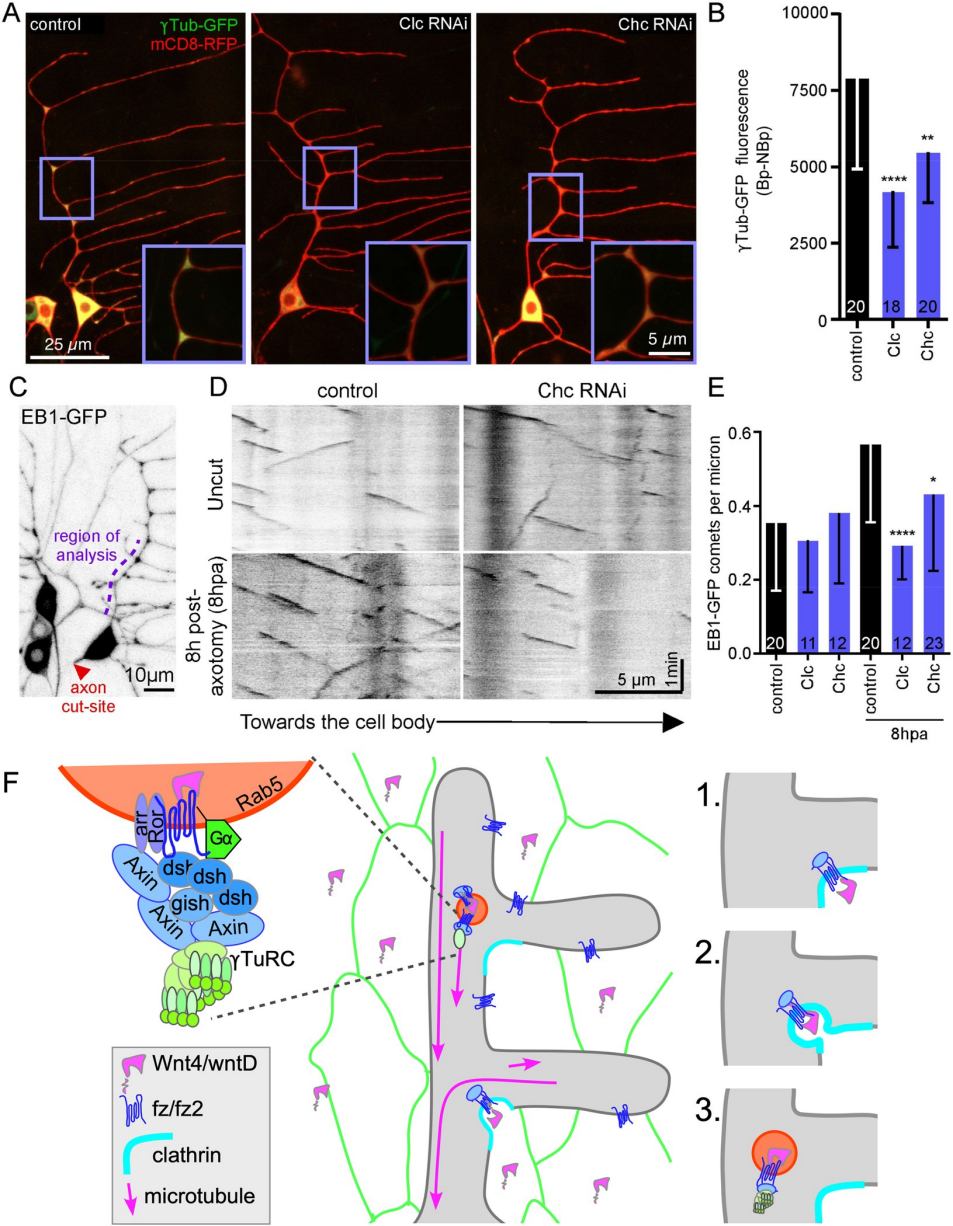
Microtubule nucleation in dendrites requires clathrin. (A) Overviews and zoomed-in insets showing γTub localization in ddaE dendrites expressing UAS-γTub-GFP, UAS-mCD8-RFP, and the UAS-RNAi under the 221-Gal4 driver. (B) Quantitation of average γTub-GFP fluorescence in different conditions. The numbers on the bar graph represent the total number of neurons. **, *P*< = 0.01, ****, *P*< = 0.0001 with Mann–Whitney test. (C) Overview of a ddaE neuron expressing UAS-EB1-GFP under the 221-Gal4 driver. The region of analysis is marked in blue and red arrowhead marks the site of axon injury. (D) Representative kymographs of EB1-GFP comets from both Uncut and 8 h post-axotomy conditions. (E) Quantitation of the number of EB1-GFP comets per micron in different conditions. The numbers on the bar graph represent the total number of neurons analyzed. *, *P*< = 0.05, ***, *P*< = 0.001, and ****, *P*< = 0.0001 with Mann–Whitney test; data values are in S2 Table. (F) Model for generating endosomal nucleation sites at dendrite branch points. The diagram at left includes the players previously identified as acting upstream of microtubule nucleation at branch points. In the middle Wnts are shown being generated in skin cells next to neurons and fz receptors are throughout the dendrites. At the right is a step-wise model for how Wnt receptors (1) concentrate at clathrin patches and can recruit Axin and dsh, (2) undergo endocytosis, and (3) either mature into or fuse with Rab5 endosomes that are then able to nucleate microtubules.

After establishing a relationship between clathrin-mediated endocytosis and apocryphal Wnt signaling proteins at dendrite branch points, we wanted to probe the effect of altering endocytosis on microtubule nucleation. As for dsh-GFP, reducing Clc and Chc by RNAi reduced the fluorescence of γTub-GFP at dendrite branch points (Fig 8A and 8B), while there was no effect on fluorescence in the dendrite trunk between branch points (S6E Fig). Cell body reduction of γTub-GFP was seen for Clc RNAi, but we did not observe a strong reduction with Chc RNAi (Figs 8A and S6D). To test the functional impact of changes in endocytosis on microtubule nucleation, we again assayed injury-induced microtubule nucleation (Fig 8C). No significant difference in microtubule dynamics was observed in uninjured neurons expressing Clc or Chc RNAi (Fig 8D and 8E). However, these conditions reduced the ability of neurons to increase microtubule plus end number in response to axon injury (Fig 8D and 8E). In all cases Clc RNAi had a stronger effect than Chc RNAi consistent effects on RFP-Clc (S5 Fig). Taken together with the change in dsh when endocytosis is disrupted and the effect of overexpression of Wnts in skin cells on branch point microtubule spawning, this data suggests that local endocytosis of Wnts at dendrite branch points acts upstream of microtubule nucleation (Fig 8F).

## Discussion

The identification of Wnt signaling endosomes as microtubule nucleation sites at dendrite branch points (Weiner and colleagues) [24] raised the possibility that neighboring cells could influence neuronal microtubule nucleation through Wnt secretion. We used a combination of in vivo live imaging and genetics to identify skin cells as a source of 2 Wnt ligands (Wnt4 and wntD) that act upstream of branch point microtubule nucleation (Fig 8F). Multiple readouts of dendritic microtubule nucleation including localization of γTub-GFP and the increase in microtubule dynamics in response to axon injury were used to confirm the role of these Wnt ligands.

While Wnt ligands were required to increase microtubule dynamics in response to axon injury, there was no change in microtubule dynamics in uninjured neurons. Does this mean that this pathway is only active in stressed neurons? Let us start with microtubule nucleation itself; does it occur in dendrites of uninjured neurons? As for Wnt ligands, even strong loss of function for γTubulin (null/hypomorph mutant animals) does not result in reduced microtubule dynamics in ddaE dendrites [14], nor is number of growing microtubules reduced in *centrosomin* (cnn, the major activator of nucleation) mutants [26]. Does this mean that nucleation itself is not important in uninjured ddaE dendrites? Microtubule polarity is disrupted in ddaE dendrites in null/hypomorph *γTubulin* mutants indicating a baseline role for nucleation at minimum in controlling microtubule polarity. Moreover, γTubulin localizes to dendrite branch points in uninjured neurons, and initiation of new microtubules is concentrated at branch points [14]. These cells seem either to have excess nucleation capacity that is regulated at baseline and/or compensatory mechanisms that can maintain microtubule number when levels of γTubulin are reduced. In support of the first possibility, kinetochore proteins act as constitutive inhibitors of microtubule nucleation in uninjured neurons [18]. In support of the second possibility, microtubule nucleation acts in parallel with and can be compensated for by other microtubule minus end regulators in *C*. *elegans*, and strong microtubule defects are observed only when both are reduced together [71]. It seems most likely that microtubule nucleation does occur in uninjured ddaE dendrites, but that microtubule dynamics is quite robust to reduction of nucleation. With this backdrop, we can now look at whether Wnt signaling acts upstream of nucleation at branch points in uninjured neurons. We previously showed that: (1) reduction of Wnt signaling proteins including fz, fz2, dsh, and Axin reduces γTubulin-GFP localization to dendrite branch points; (2) that fz, dsh, and Axin all localize to puncta at branch points that are sites of new microtubule initiation; and (3) that reduction of any of these proteins results in mixed microtubule polarity in dendrites. All of this data suggests that the Wnt signaling pathway acts upstream of branch point nucleation in uninjured neurons. Here, we use the same γTubulin localization assay that we previously used to identify Wnt receptors to identify Wnt4 and wntD as potential ligands. We show that Wnt4 is indeed expressed in neighboring skin cells at baseline. We then go on to show that expressing either one of the Wnts identified in our screen, but not a different Wnt, can increase microtubule dynamics in dendrites. It is difficult to imagine that we could have identified the correct ligands using a screen in uninjured neurons if this pathway was not active at baseline. Modulation of this pathway is important for neuronal injury responses including neuroprotection [3] and both of the ligands we identified in the screen are required to up-regulate microtubule dynamics after axon injury. Thus, we think that this pathway is active in healthy mature neurons but becomes particularly important as they respond to stress. One remaining point to note is that the phenotype from Wnt4 or wntD reduction is weaker than that previously described for knockdown of the downstream players in the pathway. For example, neither Wnt4 nor wntD RNAi resulted in mixed microtubule polarity in dendrites (S1 Fig), whereas knockdown of fz, fz2, dsh, and Axin did [24]. It seems unlikely that the pathway would be only partially dependent on ligand; therefore, a more parsimonious explanation is that the 2 ligands are somewhat redundant, and expression of either one can support some pathway function. This interpretation is supported by the overexpression result, which demonstrated that overexpression of a single ligand could up-regulate nucleation at branch points.

It is unclear why 2 Wnt ligands, 2 frizzled receptors, and 2 co-receptors are required for dendritic microtubule nucleation. It is tempting to speculate that each Wnt binds a specific receptor/co-receptor pair. Indeed, Wnt4 and wntD are quite different; wntD is the only Wnt thought not to be lipid-modified as it does not have the conserved serine residue that gets lipidated by porcupine (acyltransferase) and its secretion does not depend on Wntless [72]. However, increasing expression of either Wnt4 or wntD, but not wg, in skin cells is sufficient to increase microtubule dynamics in dendrites suggesting total amount of theses Wnt ligands controls nucleation. Wg was chosen for comparison as it has previously been shown to be expressed in the larval epidermis and reported to influence the direction of ddaE branching [73]. While there is cross-reactivity between Wnts and receptors [30,32], there is specificity in this system as did not have the same effect as the 2 Wnts we identified in our screen.

The fact that both reduction and overexpression of Wnt ligands in skin cells impacts neuronal microtubule dynamics suggests a regulatory role of skin cells in nucleation. This non-cell-autonomous control of the neuronal microtubule cytoskeleton is surprising. When neuronal cell culture was first developed, one of the key observations was that neurons can polarize correctly without contact with other cells [74]. As dendrite polarity requires correct organization of the microtubule cytoskeleton [9,75,76], it seemed a reasonable assumption that something as fundamental as nucleation would be cell-autonomous. Instead, at least for *Drosophila* sensory neurons, nucleation can be influenced by surrounding cells. Why would this be the case? Axon injury and other types of stress trigger an increase in microtubule nucleation that helps the neuron resist degeneration [3], and the increase after axon injury depends on Wnt secretion from skin cells (Fig 3). So, one possibility is that stabilization in response to neuronal stress is regulated by increased Wnt secretion from surrounding cells. However, it is also possible that the key regulatory step happens within the neuron itself, indeed we know that it requires neuronal transcription through fos [16], so reduced Wnt secretion might simply be permissive in this case rather than instructive. Alternatively, Wnt secretion from surrounding cells might increase nucleation, and thus neuroprotection, in response to more global challenges. For example, it would make sense for neurons to turn on a stabilization response if skin cells were damaged. In future studies, it will be interesting to identify a biological context in which changes in Wnt secretion from skin cells modulate neuronal microtubule nucleation.

One aspect of microtubule nucleation in dendrites that is intriguing is why it occurs at dendrite branch points. Dendrite branch points are almost like mini cell bodies as they contain ribosomes [43], Golgi [77], endosomes [70], and mitochondria [78] in addition to nucleation sites. One possibility is that branch points are a default dumping ground. However, the fact that Wnt signaling proteins are required to position the cytoskeletal regulator Apc2 [78] and nucleation sites to branch points suggests that localization is actively controlled. In this study, we identified endocytosis as the key step in Wnt signaling that confers spatial patterning. We find that endocytic sites are strongly concentrated at dendrite branch points and that alterations in endocytosis disrupt localization of Wnt signaling proteins and nucleation sites to branch points. Little is known about patterning of endocytic sites in cells, so one can only speculate about why branch points are favored for clathrin puncta localization. Membrane tension has been shown to globally regulate levels and speed of endocytic vesicle formation in cell cultures [79–82]. So, it is possible that regional differences in membrane tension could control spatial distribution of endocytosis. One other scenario where endocytosis has been localized to a specific region of the dendrite occurs during pruning of sensory dendrites in *Drosophila* pupae. Many dendritic arborization neurons are pruned during pupariation and then new dendrites innervate the adult body wall [83]. During pruning, the dendrites become disconnected at the base, and this is associated with high levels of endocytosis in dilated membrane varicosities in this region [84]; the wider varicosities would be expected to have lower membrane tension than narrow tubes, similar to wide dendrite branch points.

One unexpected feature of clathrin puncta localized to branch points was how long-lived they are, with some persisting for an entire 90-min imaging period. In many experiments, disappearance of a spot of clathrin at the plasma membrane is used as a readout of endocytosis [85,86]. However, a previous study of clathrin dynamics in dendrites of cultured hippocampal neurons found that patches became much longer-lived as neurons matured. In dendrites of young neurons, patches typically disappeared within 2 min, while average patch duration increased to about 20 min in more mature neurons [87]. In this study, clathrin patches at the membrane did not move, but moving spots emerging from these static ones were observed and were interpreted as budded vesicles in the process of uncoating [87]. If we interpret our data in the same way, then the long-lived puncta at dendrite branch points represent patches of clathrin that likely give rise to endocytic vesicles, and the dynamic spots that disappear when scission is blocked by incubating *shi*^*ts1*^ animals at the non-permissive temperature are newly endocytosed vesicles. In cultured hippocampal neurons, clustering of endocytic sites at branch points was not observed [87]. Possible explanations for this difference include: (1) hippocampal neurons were studied in culture rather than in vivo; (2) hippocampal neurons are much larger than ddaE neurons and so may require more endocytic sites; or (3) hippocampal neurons are postsynaptic and spines may replace branch points as preferred endocytic sites. It will be interesting to see where endocytic sites localize on other types of neurons in vivo.

The colocalization of Wnt signaling proteins with clathrin patches at branch points is reminiscent of signalosome formation at the plasma membrane in other cell types [88,89]. While some studies have suggested plasma membrane signalosomes are active for Wnt signaling, others have demonstrated endocytosis of signaling complexes is required [33,58]. We previously observed new microtubules emerging from mobile Wnt signaling puncta and Rab5 endosomes [24]. Our current data is also consistent with endocytosis playing a positive role in localization of active nucleation sites to dendrite branch points.

In summary, we demonstrate that surrounding cells modulate microtubule nucleation at dendrite branch points through secretion of Wnt ligands. Wnts are required to increase nucleation in response to injury, suggesting this pathway could promote neuronal stability in stress conditions. We also show that endocytic sites in dendrites are localized at dendrite branch points, and this regional specialization patterns local nucleation at branch points. The relationship between endocytosis and microtubule nucleation is unexpected, perhaps in part because microtubule nucleation has been studied mechanistically primarily in vitro either in solution or cultured cells. It will be intriguing to determine whether surrounding cells pattern nucleation or influence its activity in other contexts and whether this also occurs through Wnt signaling.

## Materials and methods

### *Drosophila* fly stocks and maintenance

Fly lines were grown at 25°C in plastic bottles or vials containing fly food except for the heat-sensitive *shi*^*ts1*^ (grown at 20°C). Fly food components included cornmeal, yeast, dextrose, sucrose, agar, tegosept, and propionic acid. RNAi lines were requested either from Bloomington Drosophila Stock Center (BDSC) or Vienna Drosophila Resource Center (VDRC). The details of all the fly lines used are listed in S1 Table. The control-RNAi line used in all the experiments is γTub37c-RNAi (isoform of γTub that is maternally deposited and not expressed in neurons). The other control lines are described in the corresponding figure legends.

Crosses for experiments were made by mixing virgin females from the tester lines and with males from RNAi, mutant, or overexpression lines. The crosses were placed at 25°C to collect embryos daily on food caps and further aged to obtain 3-day-old larvae for imaging. The genotypes of the tester lines used in this study are listed below:

Figs 1B, 1H, 1I and 2A and 2B: TMC-QF, QUAS-myr-tdTomato, QUAS- γTub-GFP/CyO; A58-Gal4, dicer2-nls-BFP/TM6
Figs 1C, 1F and 1G: TMC-QF, QUAS-myr-tdTomato, QUAS-γTub-GFP/CyO; repo-Gal4, UAS-dicer2-nls-BFP, UAS-iBlueberry/TM6
Fig 1D and 1E: TMC-QF, QUAS-myr-tdTomato/CyO
Fig 2C: TMC-QF, QUAS-myr-tdTomato, QUAS-γTub-GFP/CyO; *wntD*^*KO2*^/TM6
Fig 2D: *Wnt4*^*EMS23*^/CyO; 221-Gal4, UAS-γTub-GFP/TM6, and *Wnt4*^*C1*^/CyO; UAS-mCD8-RFP/TM6
Fig 3: TMC-QF, QUAS-EB1-GFP/CyO-tb; A58-Gal4, dicer2-nls-BFP/TM6-sb,tb
Fig 4: 221-Gal4, UAS-mCD8-RFP/TM6 and TMC-Gal4, UAS-RFP-Clc/CyO
Fig 5: UAS-GFP-Clc/CyO; 221-Gal4, UAS-EB1-TagRFP-T/TM6
Fig 6A: 221-Gal4, UAS-fz-GFP/TM6 and TMC-Gal4, GFP-Clc/CyO
Fig 6B: 221-Gal4, UAS-Rab5-GFP/TM6 and 221-Gal4, UAS-dsh-GFP/TM6
Fig 6B and 6C: 221-Gal4, UAS-Axin-GFP/TM6
Fig 7A and 7B: UAS-dicer2, UAS-mCD8-RFP/CyO; 221-Gal4, UAS-dsh-GFP/TM6
Fig 7C and 7D: UAS-dicer2, UAS-mCD8-RFP/CyO; 221-Gal4, UAS-Rab5-GFP/TM6
Fig 8A and 8B: UAS-dicer2, UAS-mCD8-RFP/CyO; 221-Gal4, UAS-γTub-GFP/TM6
Fig 8C–8E: 221-Gal4, UAS-EB1-GFP, UAS-dicer2-nls-BFP/TM6.

### Construction of plasmids and fly lines

The following materials were used for cloning: Herculase (600679) from Agilent was used as polymerase for PCR and primers were synthesized by IDT. Restriction enzymes, T4-DNA Ligase (M0202), and Quick-CIP (M0525) were from New England Biolabs. DNA fragments were purified from agarose gels using a NucleoSpin kit from Takara (740609.250). Cloning steps were carried out using either T4 Ligase or an In-Fusion kit from Takara (638944). Site-directed mutagenesis was carried using a Q5 kit (NEB E0554S). DNA synthesis was performed by GeneWiz.

To convert to from UAS to 4X QUAS, the pUAST-γTub-GFP construct [14] was digested with restriction enzymes SphI and BglII and a 2,771 bp fragment containing the 5X-UAS-hsp70 minimal promoter was gel isolated and column purified for cloning into SphI-BglII digested 1GCT-pHD-dsRed backbone (derived by Chengye Feng from pHD-dsRed; [90]) for site-directed mutagenesis using the Q5 kit. The following primers were used to delete the 5X-UAS sequences and introduce a unique PacI restriction site upstream from the hsp70 minimal promoter:

Q5PacIF1: TTAACGGAGACTCTAGCGAGCGCCGGAGTATAAATAG
Q5PacIR1: TTAAACCTGCAGGCATGCGAATTCCACCTGCTTCAG.

The resulting vector was digested with PacI and then gel isolated and column purified. The linearized vector was treated with Quick-CIP, followed by heat inactivation in preparation for ligation. Ligation was then carried out with a 112 bp PacI restriction fragment isolated from QF2-QUAS-E1B-nls-Cardinal [91] using standard cloning methods. Clones were characterized by restriction digest and sequencing to determine the orientation of the fragment. To generate pQUAS-γTub-GFP, the SphI-BglII fragment with the newly introduced QUAS sequence was subcloned back into the SphI-BglII backbone of pUAST-γTub-GFP.

To generate pQUAS-EB1-GFP, the pUAST-EB1-tagRFP-T construct [92] was used as template to amplify the Drosophila EB1 open reading frame using the following primers:

EB1XhoIF1: CAGATCTGCGGCCGCGGCTCGAGGCCACCATGGCTGTAAACGTCTACTCCACAAATGTG
EB1NheIR1: CTCATACCTCCACTACCGCTAGCATACTCCTCGTCCTCTGGTGGTGCATCGTCAG

The EB1 PCR fragment was then gel isolated and column purified. The construct pQUAS-γTub-GFP was digested with the restriction enzymes XhoI and NheI and the vector backbone gel isolated and column purified. The EB1 PCR fragment was then cloned into the backbone using In-Fusion to produce pQUAS-EB1-GFP.

To generate pQUAS-3X-cyto-BFP, the following primers were used to PCR amplify 3X-cyto-BFP using pHAGE-TO-nls-st1dCas9-3nls-3XTagBFP2 as the template [93]

3XBFPBglIIF1: GGGAATTCGTTAACAGATCTCGGTCGCCACCATGGCCTCCTCCGAGGACGTCGG
3XBFPXbaIR1: CCTTCACAAAGATCCTCTAGATACAGGAACAGGTGGTGGCGGCCCTCGGCG

The PCR product was then cloned by In-Fusion into the BglII-XbaI digested backbone of pQUAS-γTub-GFP.

CRISPR-Wnt4-1xmNG strategy:

The 5′ and 3′ gRNA target sites were selected in the first intron and 3′ UTR of wnt4, respectively, using the online resource CRISPR Optimal Target Finder [90]

Wnt4 CRISPR 5′ target: GGGCAGTACAAACTCTGAATAGG
Wnt4 CRISPR 3′ target: TCAATATTCTTAGCTCTAGAAGG

To make the pCFD5-Wnt4-guide construct the following primers were used for PCR amplification with pCFD5 as template:

wnt4gF1: GCGGCCCGGGTTCGATTCCCGGCCGATGCAGGGCAGTACAAACTCTGAATGTTTTAGAGCTAGAAATAGCAAG
wnt4gR1: ATTTTAACTTGCTATTTCTAGCTCTAAAACTCTAGAGCTAAGAATATTGATGCACCAGCCGGGAATCGAACCC

The gel purified PCR fragment was then cloned into BbsI digested pCFD5 to generate pCFD5-Wnt4-guide following the protocol of [94].

To generate the repair template for wnt4 the CRISPR targeting vector 1GCT-pHD-dsRed (derived by Chengye Feng from pHD-dsRed; [90] was first modified by replacing GFP with 1xmNG. To do this the following primers were used to amplify 1XmNG using EB3-3xmNeonGreen-N1) as template:

BmtI1XmNGF1
CCACGCGTGGCCGGCCTGCTAGCGCCACCATGGTGAGCAAGGGCGAGGAGG
Eco53kI1XmNG
GACTCTGCGATCGCTATGAGCTCTTGTACAGCTCGTCCATGCCCATCTCATCG

The resulting PCR product was gel purified and cloned by In-Fusion into the backbone of BmtI-Eco53kI digested 1GCT-pHD-dsRed to generate 1GCT-1XmNG-pHD-dsRed.

To generate 1GCT-Wnt4-1XmNG-pHD-dsRed the following primers were first used to PCR amplify Wnt4 sequences upstream of the stop codon from *D*. *melanogaster* genomic DNA:

SrfIF1
AAGACTGGGCCTTTCGCCCGGGCTCTCATGGAGTAAAGATCTGCAGATCGACACATG
SphIR1
CGCGTGGTACCGCCGGCTGCTTTACAAAAGTATTTGTTCTCCAGCCGC

The PCR product was then cloned by In-Fusion into SrfI-SphI digested 1GCT-1XmNG_pHD-dsRed to produce 1GCT-Wnt4-1XmNG_pHD-dsRed-S1.

The following primers were then used to PCR amplify the 5′ homology arm of wnt4 from *D*. *melanogaster* genomic DNA:

5ArmF1
AAGACTGGGCCTTTCGCCCGAAATCGACTGGAGAGTTGGTAGCCCAATGCAAG
5ArmR1
CTTTACTCCATGAGAGCCCCGTCCAAAACCCTTGGGGTCTTCAATTACAG

The PCR product was then cloned by In-Fusion into SrfI digested 1GCT-Wnt4-1XmNG-pHD-dsRed-S1 to produce 1GCT-Wnt4-1XmNG-pHD-dsRed-S2.

The terminator sequence of Wnt4 was amplified using the following primers from *D*. *melanogaster* genomic DNA:

AsiSIF1
TGTACAAGAGCTCATAGCGTAGACGGGGTGCCATCTCTCGCAGACTTTCTTTGCC
AsiSIR1
GCCGCGATGTCGACTCTGCTACATTCAATTTAGTAGTAAATAAATTCGACTG

The PCR product was then cloned by In-Fusion into AsiSI digested 1GCT-Wnt4-1XmNG-pHD-dsRed-S2 to produce 1GCT-wnt4-1XmNG-pHD-dsRed-S3.

The 3′ homology arm of Wnt4 was synthesized by GeneWiz as a 1525 bp fragment with terminal AscI sites. The synthesized product was AscI digested and gel purified for cloning into AscI digested.

Quick-CIP treated 1GCT-Wnt4-1XmNG-pHD-dsRed-S3 to generate the final construct 1GCT-Wnt4-1XmNG-pHD-dsRed.

The plasmids were sent to BestGene for injection into fly embryos. The fly lines with the insertions were mapped to identify which chromosome has the insertion using balancers (except for CRISPR/Cas9-mediated tagging of Wnt4). Standard methods in genetics were further used to construct tester lines for different experiments.

### Confocal imaging of *Drosophila* larvae and image processing

Three-day-old larvae were removed from a 35 mm food cap and rinsed in water for mounting. Larvae were mounted with dorsal side up and immobilized on a slide coated with dry 3% agarose by gently pressing down a coverslip and holding it in place with tape. Images were acquired on Zeiss LSM800 microscopes with Plan Apochromat 63× oil immersion objectives the Zen Blue microscope software and GaAsP detectors except where Airyscan is specified. The 10× objective was used to identify and focus specifically on the ddaE neurons or cells present in the A4 or A3 segment of the larva. The Airyscan detector and processing on the LSM800 was used to acquire images in S2 Fig. Two neurons were imaged per larva for most of the imaging experiments except the EB1-GFP movies and *shi*^*ts1*^ experiments where one neuron was imaged per larva. Fiji/ImageJ software was used to process and quantitate all the images and movies.

### Imaging and quantitating EB1-GFP dynamics post-axotomy

For axotomy, a manual MicroPoint laser system (Andor Technology) was used to sever the axon at its base and the larva was recovered from the agarose slide and transferred to petri dishes with fly food and incubated at 20°C for 8 h before imaging.

For Fig 3, EB1-GFP dynamics was imaged using a Zeiss AxioImager M2 microscope with a Zeiss 506 CMOS camera. The recordings were taken for 300 frames (5 min) with a frequency of 1 frame per second. For Fig 8, EB1-GFP dynamics was acquired on an LSM 800 microscope for 250 frames with a frequency of 1 frame per 1.27 s. The movies were processed using the bleach correction and template matching tools on ImageJ before quantitation. The region of interest (ROI) for counting EB1-GFP comets included the in-focus region between the second branch point and fifth/sixth branch point of the dorsal comb dendrite of ddaE. The region between cell body and first branch point was not included in the ROI as it typically tends to have more lattice binding of EB1-GFP, making it hard to quantitate the number of comets. The number of EB1 comets was manually counted and divided by the corresponding length of the ROI to obtain EB1-GFP comets/micron for each neuron. The number of comets was also confirmed with kymographs of each video acquired by using the Multi Kymograph tool on ImageJ (line width of 1, 3, or 5 was used). “Spawning” events were assessed in the 300 frame movies described above. The formation of a new EB1-GFP comet was termed as a spawning event. The absence of comet in the region before (few frames) it originated was used to classify it as a new comet. Only the regions of the neuron in complete focus were considered for quantitation. A plus end comet growing off a minus-end-out comet was not considered a spawning event. Only spawning events in the main trunk of the dorsal ddaE dendrite were considered and these were classified as occurring at branch points or between branch points. The numbers reported are the total number of events in the in focus region of the main trunk in the 300 frame movie.

### Quantitation of fluorescence intensity and occupancy at branch points

For measuring intensity of γTub-GFP, Z-stack images of each neuron were projected using the maximum intensity tool in Fiji. Fiji’s polygon selection tool was used to draw an ROI around the branch point (Bp) and non-branch point (nBp) regions of the dendrite’s main dorsal trunk to collect mean fluorescence intensity of γTub-GFP in these regions. For each neuron, the average Bp-nBp intensity was calculated from multiple Bp and nBp regions. The same approach was used to calculate the intensity of cyto-BFP.

For these measurements only branch points along the main trunk of the dorsal dendrites were used. The triangular junction was outlined (see Fig 1H) and could include up to 0.5 microns of taper into the cylindrical non-branch point.

For calculating occupancy of GFP-Clc or RFP-Clc, AP-2α-GFTSTF and Rab5-GFP, the presence or absence of puncta was checked (any puncta above background fluorescence was counted), and the fraction calculated by taking the ratio between the number of branch points occupied by puncta and total number of branch points of main trunk per image/neuron. As for γTub-GFP measurements, the triangular branch point and up to 0.5 microns of taper at the border into the cylindrical dendrite was defined as the branch point. When 2 branch points appeared fused, they were considered as 2 separate branch points and the occupancy was quantitated for each branch point.

For calculating occupancy of dsh-GFP, since there were multiple types of puncta with varying intensity, we considered a branch point occupied for dsh-GFP if it had an average fluorescence greater than 5,000. The same ratio and method described above was used to calculate the fraction. For measuring intensity of dsh-GFP and Rab5-GFP, a circular ROI in Fiji was used to measure the fluorescence values of the puncta.

### Rearing and imaging *shi*^*ts1*^ larvae

In Fig 5, we used *shibire*^*ts1*^, a heat-sensitive mutant allele of dynamin in flies. Virgin females from *shi*^*ts1*^ line were crossed with males from the tester line. We leveraged *shi*^*ts1*^ being on X chromosome as we were able to use male and female progeny of the cross to image *shi*^*ts1*^/O and *shi*^*ts1*^/+, respectively. Gonad identification and paralysis due to heat shock was used to segregate the gender of the larvae. Before imaging the larvae, they would be placed in the 37°C (restrictive temperature for *shi*^*ts1*^) incubator for 15 min (Fig 5A and 5B) or 45 min (Fig 5C and 5D). The incubation time was increased to 45 min for FRAP experiments to make sure we observed the maximum effect of the allele. After the pre-incubation, larvae were imaged on the LSM800 confocal microscope using a Bioptechs objective heating collar set to 37°C.

### Fluorescence recovery after photobleaching

FRAP was performed using the bleaching module of the Zen Blue software on the Zeiss LSM800 confocal microscope. Stationary GFP-Clc at branch point of ddaE neurons were bleached and recovery within 90 s was monitored using time-lapse imaging. Proximal branch points, position 2 to 5 from the cell body, were used for the assay. For quantitation, the ROI was marked around the stationary puncta and the Time Series Analyzer in ImageJ was used to measure the fluorescence values of the puncta over time. These raw fluorescence values were normalized using the formula (Current value–minimum value)/(maximum–minimum value). The minimum value for each data set was set to the fluorescence value post-bleaching. Normalized fractional recovery post-bleaching was plotted against time to visualize differences in GFP-Clc exchange across each group.

### Long-term imaging of GFP-Clc dynamics

In order to immobilize and image the larva for a longer period of time, we used the LarvaSPA method from Chun Han’s lab [64]. In summary, the larva was immobilized on a coverslip with double sided tape, PDMS bridge and optically clear UV glue such that its body segments (A2 to A5) were stuck to the coverslip while the head and tail were free to move. This allowed the larva to breathe and stay alive longer while the middle part of its body is immobilized. Only 1 larva was mounted in each coverslip. The coverslip was placed in a customized chamber that had a few drops of water on a Kimwipe to keep the larva moist. A Z-stack covering the ddaE neuron was acquired using the 63× objective on the LSM800 upright microscope once in 3 min over the 90 min of time-lapse imaging of GFP-Clc. The stack was processed with each time point as a maximum projection on ImageJ to generate the movie (S4 Movie).

### Quantitation of fluorescence intensity in the cell body

For dsh-GFP maximum projection images of Z-stacks acquired at 0.5 zoom with a 63× objective on an LSM 800 microscope were used for quantitation. The polygon tool in ImageJ was used to draw an outline of the cell body and the raw fluorescence values from the green channel (dsh-GFP/γTub-GFP) were recorded. The nucleus was included in the quantitation.

For γTub-sfGFP (endogenous tagged γTubulin) males expressing UAS mCD8-RFP were crossed to female virgins from the line: γTub-sfGFP; 221Gal4, UAS-iBlueberry, UAS-dicer2-nls-BFP. For each larva, the axon of the sixth ddaE neuron on one side of the body (from the tail end) was severed using a MicroPoint pulsed UV laser; 24 h post severing the neuron with the severed axon was imaged as was the contralateral spared cell, which served as control. Acquired images were quantified in FIJI using a polygon selection around the cell body and an oval around the nucleus. Due to high background noise, single slice from a z-stack was used. Cell body florescence was calculated as: total fluorescence in the cell body–total fluorescence in the nucleus/area of cell body–area of nucleus.

### Statistical methods

GraphPad Prism version 6.0 was used to perform statistical tests that are mentioned in the figure legends. The error-bars on the bar graphs represent standard deviation.

## Supporting information

S1 Fig γTub-GFP between branch points and microtubule polarity and dynamics in dendrites when wls or Wnt ligands are reduced in skin cells. (A) Overview images of matched control and *Wnt4* and *wntD* mutant neurons. Different controls were used as the lines had to be constructed with different Gal4 drivers. (B and C) Raw fluorescence values from between branch points along the main ddaE dorsal dendrite trunk are shown. The numbers on the bars are numbers of cells measured for each condition. These measurements are from the data sets used in Fig 2. *p < 0.05 with a Mann–Whitney test. (D) The percentage of EB1-comets (plus ends) that have plus-end-out polarity in the main trunk of the dorsal ddaE dendrite was quantitated from EB1-GFP movies. There is no significant difference across the different categories with Fisher’s exact test. The numbers above the bar graphs represent the number of comets quantitated in each category. (B) Representative kymographs from EB1-GFP movies that show a majority of minus-end-out EB1-GFP comets. (C) Spawning events (numbers of new comets initiating) in the region of analysis in the ddaE dorsal dendrite trunk was quantitated from EB1-GFP movies. The numbers of neurons analyzed for each condition were: control–17, Evi-RNAi–15, wntD-RNAi–13, Wnt4-RNAi–13. All error bars show standard deviation and underlying data is in S2 Table.
(TIF)

S2 Fig Fluorescence of endogenous GFP-tagged γTubulin does not change after axon injury. (A and B) Example images of neurons expressing the membrane marker mCD8-GFP and γTub-sfGFP (sfGFP coding sequence inserted into native gene locus of γTub23C) in uninjured neurons and neurons 24 h after axon injury. Single confocal planes are shown for the γTub-sfGFP channel from the cell body and dorsal comb dendrite. In these examples, a slight increase in fluorescence can be seen within dendrite branch points. (C) Fluorescence intensity of γTub-sfGFP in the ddaE somatic cytosol was measured in single confocal sections. Numbers on the bars are numbers of cells analyzed. Error bars show standard deviation. Statistical comparison was done using a Mann–Whitney test and underlying data is in S2 Table.
(TIF)

S3 Fig Localization of endogenous Wnt4-mNG and wls-GFP. (A) Airyscan image of Wnt4-mNG showing localization in skin cells. The ddaE neuron is labeled using UAS-iBlueberry with the 221-Gal4 driver. (B) Airyscan image of wls-GFP showing localization in skin cells. The ddaE neuron is labeled using UAS-iBlueberry with the 221-Gal4 driver. In A and B approximate boundaries of epithelial cells are indicated with dashed lines. Localization of Wnt4-mNG in larval areas containing glia (C) and (D) muscles. Glial cells surround the axons and there may be some Wnt4-mNG around axons; a dashed line is between axons from ddaD and ddaE. The muscle cell can be distinguished by linear organization of internal structures; these cells definitely contain Wnt4-mNG. The ddaE neuron is labeled using UAS-iBlueberry with the 221-Gal4 driver.
(TIF)

S4 Fig Long-term imaging of GFP-Clc in dendrite of ddaE. (A) Images at different time points from S4 Movie that show some GFP-Clc puncta remain stationary (yellow arrowheads) at dendrite branch points. The time-lapse movie was acquired from a larva mounted using the LarvaSPA method [64]. (B) Orange arrowhead points to a puncta that merges with the stationary one and makes the stationary one brighter. (C) Blue arrowhead points to a puncta that buds off from the stationary one and makes the stationary one dimmer.
(TIF)

S5 Fig Partial reduction of RFP-Clc at dendrite branch points in Clc and Chc RNAi neurons. (A) Example images ddaE dorsal dendrites expressing RFP-Clc and cyto-GFP with Tmc-Gal4. Control and clathrin RNA hairpin expression was also driven with 221-Gal4. Yellow arrowheads indicate branch points with RFP-Clc puncta and blue arrowheads indicate branch points without puncta. (B and C) The intensity of RFP-Clc puncta at branch points was measured and occupancy of branch points with a puncta of any intensity above background was counted. Numbers on the graphs are number of cells analyzed and one-way ANOVA was used to compare conditions. Error bars show standard deviation and underlying data is in S2 Table.
(TIF)

S6 Fig Morphology and cell body fluorescence of ddaE neurons expressing dsh-GFP with clathrin RNAi and γTub-GFP fluorescence in the dendrite trunk and cell body with clathrin RNAi. (A) Representative overview images of control, Clc, and Chc-RNAi in ddaE neurons using the 221-Gal4 driver. The neuronal plasma membrane is labeled with UAS-mCD8-RFP and UAS-dsh-GFP puncta can be seen at dendrite branch points. Clc RNAi reduced the cell body fluorescence of dsh-GFP in some cases including the example shown here. (B) Quantitation of the total number of branch points in each neuron. There is no significant difference across the different categories with a Mann–Whitney test. The numbers above the bar graphs represent the number of neurons quantitated in each category. (C) The intensity of dsh-GFP fluorescence was measured in the cell body. (D) Quantitation of γTub-GFP in the cell body in ddaE neurons expressing clathrin RNAi hairpins. Overview images are shown in Fig 8. A Mann–Whitney test was used to compare cell body fluorescence across genotypes. (E) Fluorescence values between branch points for the data set shown in Fig 8B is shown. No change was detected when clathrin levels were reduced; underlying data is contained in S2 Table.
(TIF)

S1 Table Reagent list. *Drosophila* lines, and their sources, used in this study are shown in the table.
(XLSX)

S2 Table Data and quantitation. Measurements used to generate the graphs in each figure are included in this Table. Raw image files are available upon request from the corresponding author.
(XLSX)

S1 Movie Overexpression of wntD in skin cells increases microtubule dynamics in dendrites. Time-lapse imaging of EB1-GFP comets in ddaE dendrites; images were acquired every second for 5 min. Two movies are shown side by side. In both A58-Gal4 was used to drive expression in skin cells while QUAS-EB1-GFP was driven using the TMC-QF driver in neurons to visualize EB1-GFP comets in ddaE. Skin>UAS-control-RNAi (γTub37C) is shown on the left, and skin>UAS-wntD is shown on the right.
(MP4)

S2 Movie AP2α-GFSTF colocalizes with stationary RFP-Clc puncta and not mobile RFP-Clc puncta. Movie showing endogenous AP2α-GFSTF imaged with UAS-RFP-Clc in ddaE dendrites using TMC-Gal4 to drive expression. White arrowheads mark stationary RFP-Clc patches colocalizing with endogenous AP2α-GFSTF. The red channel (Clc) is shown on the left, the green channel (AP2α) is shown on the right.
(MP4)

S3 Movie GFP-Clc dynamics at dendritic branch points of ddaE. Clathrin exists at dendritic branch points in 2 distinct populations: larger, stationary puncta and smaller, mobile puncta. The black arrows in the first few slices point to the stationary puncta. UAS-GFP-Clc was imaged in ddaE driven by the 221-Gal4 driver.
(MP4)

S4 Movie Long-term imaging of GFP-Clc in dendrite of ddaE. Time-lapse imaging of GFP-Clc in the main trunk of a ddaE dendrite using the 221-Gal4 driver. A Z-stack of the dendrite was acquired every 3 min for 1.5 h. The white arrowheads point to the GFP-Clc puncta that remain stationary over time.
(MP4)

S5 Movie Moving GFP-Clc puncta are rare in *shi*^*ts1*^animals at the non-permissive temperature. Time-lapse imaging of GFP-Clc in the main trunk of ddaE using the 221-Gal4 driver. The larvae were heat-shocked for 15 min at 37C and imaged with a heating collar on the objective to maintain restrictive conditions for the *shi*^*ts1*^ allele: While both stationary and moving puncta are present in the control—*shi*^*ts1*^/+ (left), a reduction of moving GFP-Clc puncta was observed in the *shi*^*ts1*^animals (right). The arrows point to the stationary GFP-Clc puncta.
(MP4)

S6 Movie Reduced recovery after photobleaching of GFP-Clc in *shi*^*ts1*^. Clathrin exchange was monitored by time-lapse imaging at branch points post-bleaching under normal (left) and arrested scission conditions (*shi*^*ts1*^ at the non-permissive temperature)—right. The larvae were heat-shocked for 45 min at 37C and imaged with heating collar on the objective to maintain restrictive conditions for the *shi*^*ts1*^ allele. The arrow denotes the bleaching event. The ROI for bleaching was the outline of GFP-Clc stationary puncta.
(MP4)

S7 Movie Reduced recovery after photobleaching of GFP-Clc in shiDN animals. Dominant-negative shibire (shiDN) was used to constitutively block endocytosis at its scission step (right); control animals are shown at left. GFP-Clc recovery at room temperature was monitored for 90 s post-bleaching at dendritic branch points. The arrow denotes the bleaching event. The ROI for bleaching was the outline of GFP-Clc stationary puncta.
(MP4)

S8 Movie Colocalization of Rab5-GFP and Axin-RFP. Time-lapse imaging in ddaE using the 221-Gal4 driver shows a moving UAS-Rab5-RFP puncta (labeled with arrowhead) colocalizing with UAS-Axin-GFP forming a yellow puncta.
(MP4)

## Abbreviations

APC: adenomatous polyposis coli
BDSC: Bloomington Drosophila Stock Center
Bp: branch point
Chc: clathrin heavy chain
Clc: clathrin light chain
FRAP: fluorescence recovery after photobleaching
nBp: non-branch point
PCP: planar cell polarity
ROI: region of interest
VDRC: Vienna Drosophila Resource Center
