# Supplementary figures and images for: Endocytosis of Wnt ligands from surrounding epithelial cells positions microtubule nucleation sites at dendrite branch points
Source: PLoS Biol. 2025 Jan 6;23(1):e3002973. doi: 10.1371/journal.pbio.3002973 (PMC11703082; doi:10.1371/journal.pbio.3002973)

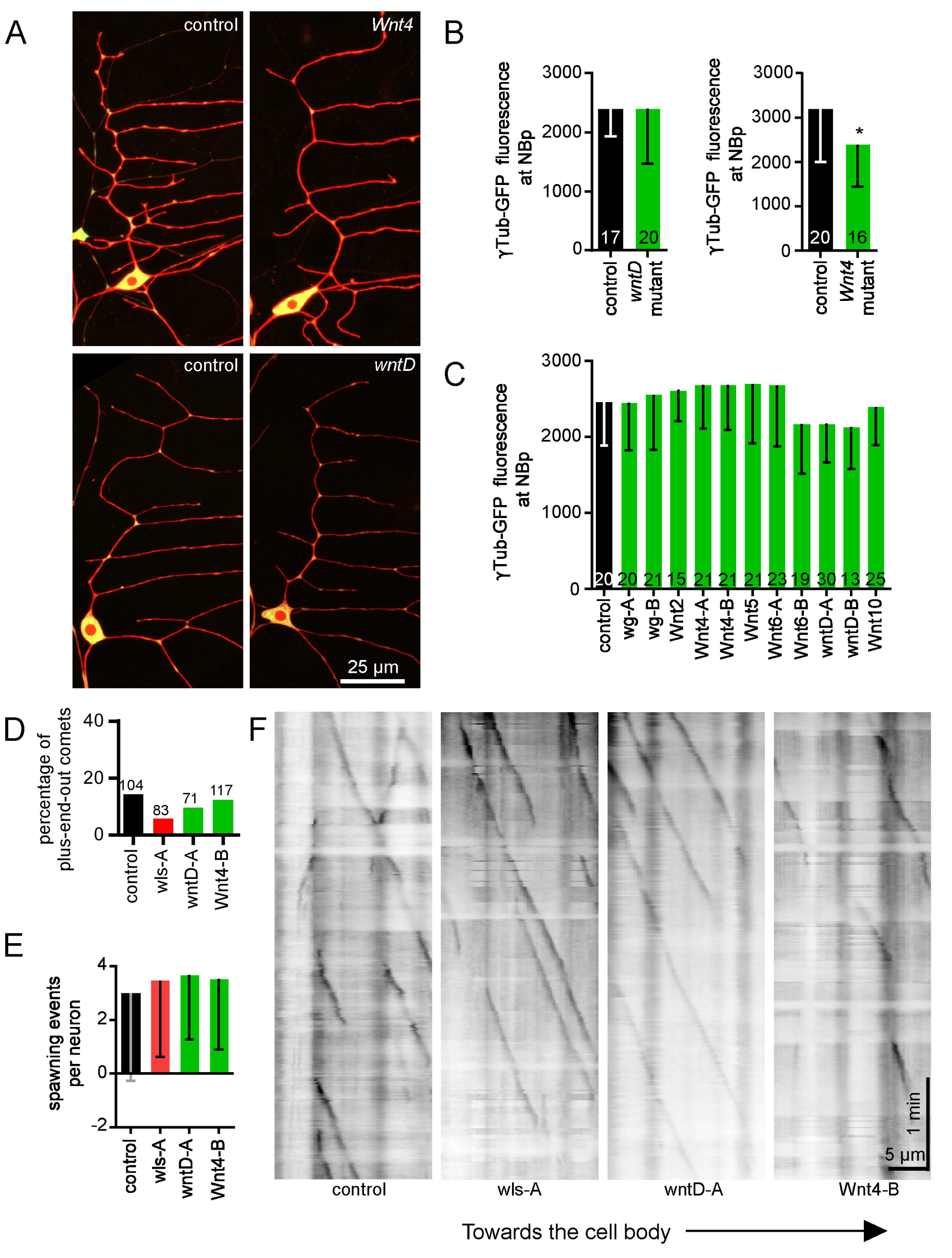

Supplement: S1 Fig — (A) Overview images of matched control and Wnt4 and wntD mutant neurons. Different controls were used as the lines had to be constructed with different Gal4 drivers. (B and C) Raw fluorescence values from between branch points along the main ddaE dorsal dendrite trunk are shown. The numbers on the bars are numbers of cells measured for each condition. These measurements are from the data sets used in Fig 2. *p < 0.05 with a Mann–Whitney test. (D) The percentage of EB1-comets (plus ends) that have plus-end-out polarity in the main trunk of the dorsal ddaE dendrite was quantitated from EB1-GFP movies. There is no significant difference across the different categories with Fisher’s exact test. The numbers above the bar graphs represent the number of comets quantitated in each category. (B) Representative kymographs from EB1-GFP movies that show a majority of minus-end-out EB1-GFP comets. (C) Spawning events (numbers of new comets initiating) in the region of analysis in the ddaE dorsal dendrite trunk was quantitated from EB1-GFP movies. The numbers of neurons analyzed for each condition were: control–17, Evi-RNAi–15, wntD-RNAi–13, Wnt4-RNAi–13. All error bars show standard deviation and underlying data is in S2 Table. (TIF) [file pbio.3002973.s001.tif]

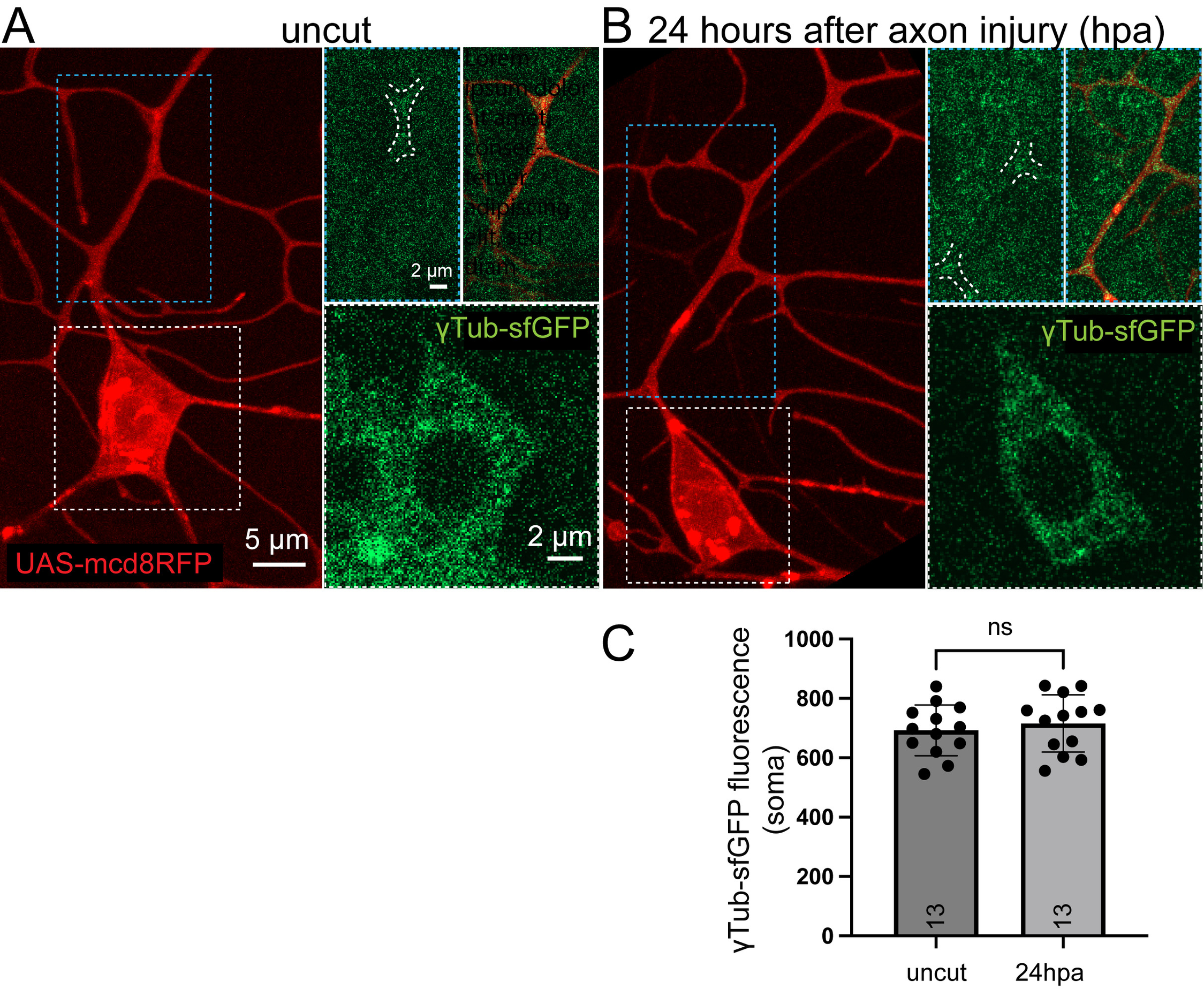

Supplement: S2 Fig — (A and B) Example images of neurons expressing the membrane marker mCD8-GFP and γTub-sfGFP (sfGFP coding sequence inserted into native gene locus of γTub23C) in uninjured neurons and neurons 24 h after axon injury. Single confocal planes are shown for the γTub-sfGFP channel from the cell body and dorsal comb dendrite. In these examples, a slight increase in fluorescence can be seen within dendrite branch points. (C) Fluorescence intensity of γTub-sfGFP in the ddaE somatic cytosol was measured in single confocal sections. Numbers on the bars are numbers of cells analyzed. Error bars show standard deviation. Statistical comparison was done using a Mann–Whitney test and underlying data is in S2 Table. (TIF) [file pbio.3002973.s002.tif]

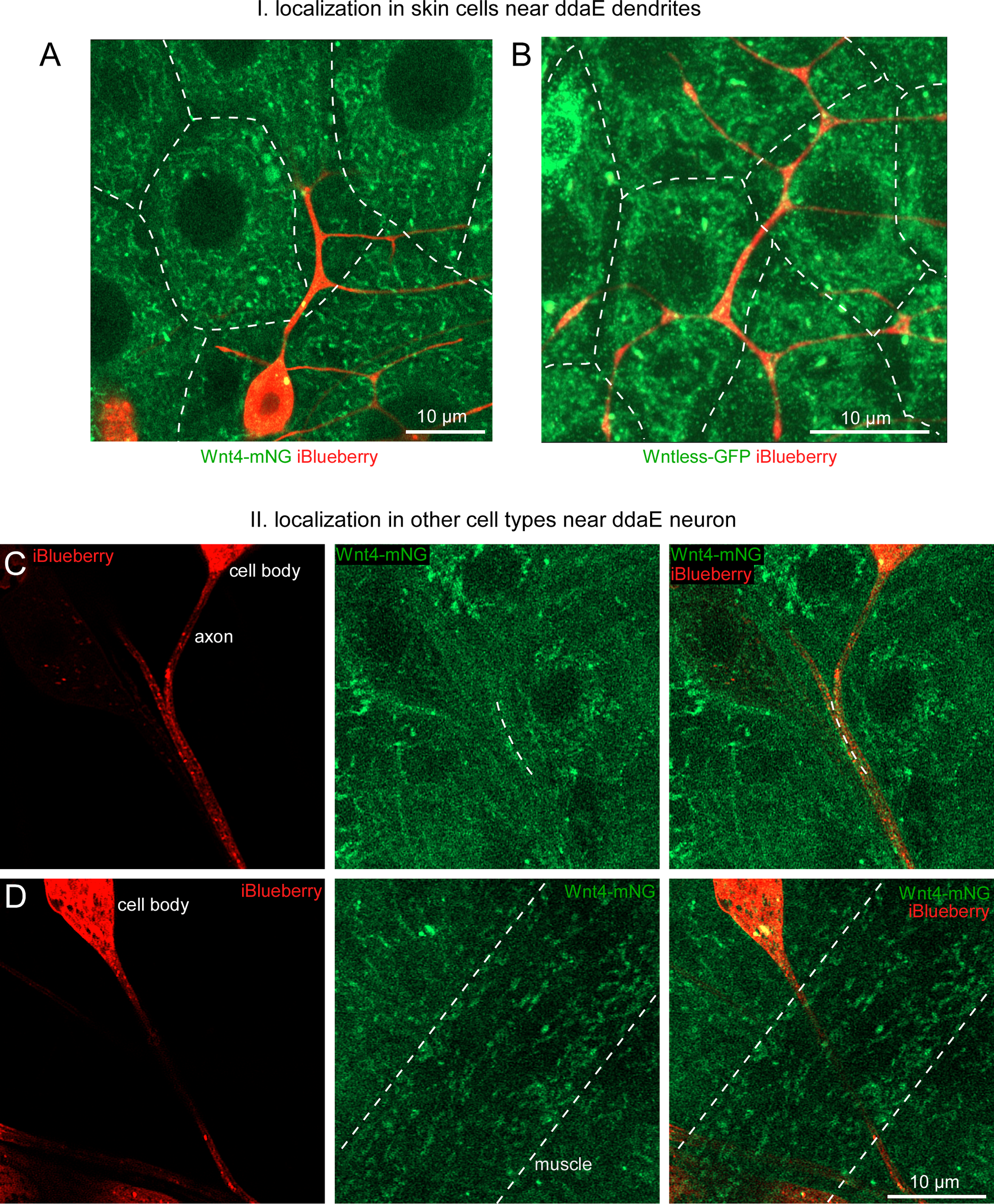

Supplement: S3 Fig — (A) Airyscan image of Wnt4-mNG showing localization in skin cells. The ddaE neuron is labeled using UAS-iBlueberry with the 221-Gal4 driver. (B) Airyscan image of wls-GFP showing localization in skin cells. The ddaE neuron is labeled using UAS-iBlueberry with the 221-Gal4 driver. In A and B approximate boundaries of epithelial cells are indicated with dashed lines. Localization of Wnt4-mNG in larval areas containing glia (C) and (D) muscles. Glial cells surround the axons and there may be some Wnt4-mNG around axons; a dashed line is between axons from ddaD and ddaE. The muscle cell can be distinguished by linear organization of internal structures; these cells definitely contain Wnt4-mNG. The ddaE neuron is labeled using UAS-iBlueberry with the 221-Gal4 driver. (TIF) [file pbio.3002973.s003.tif]

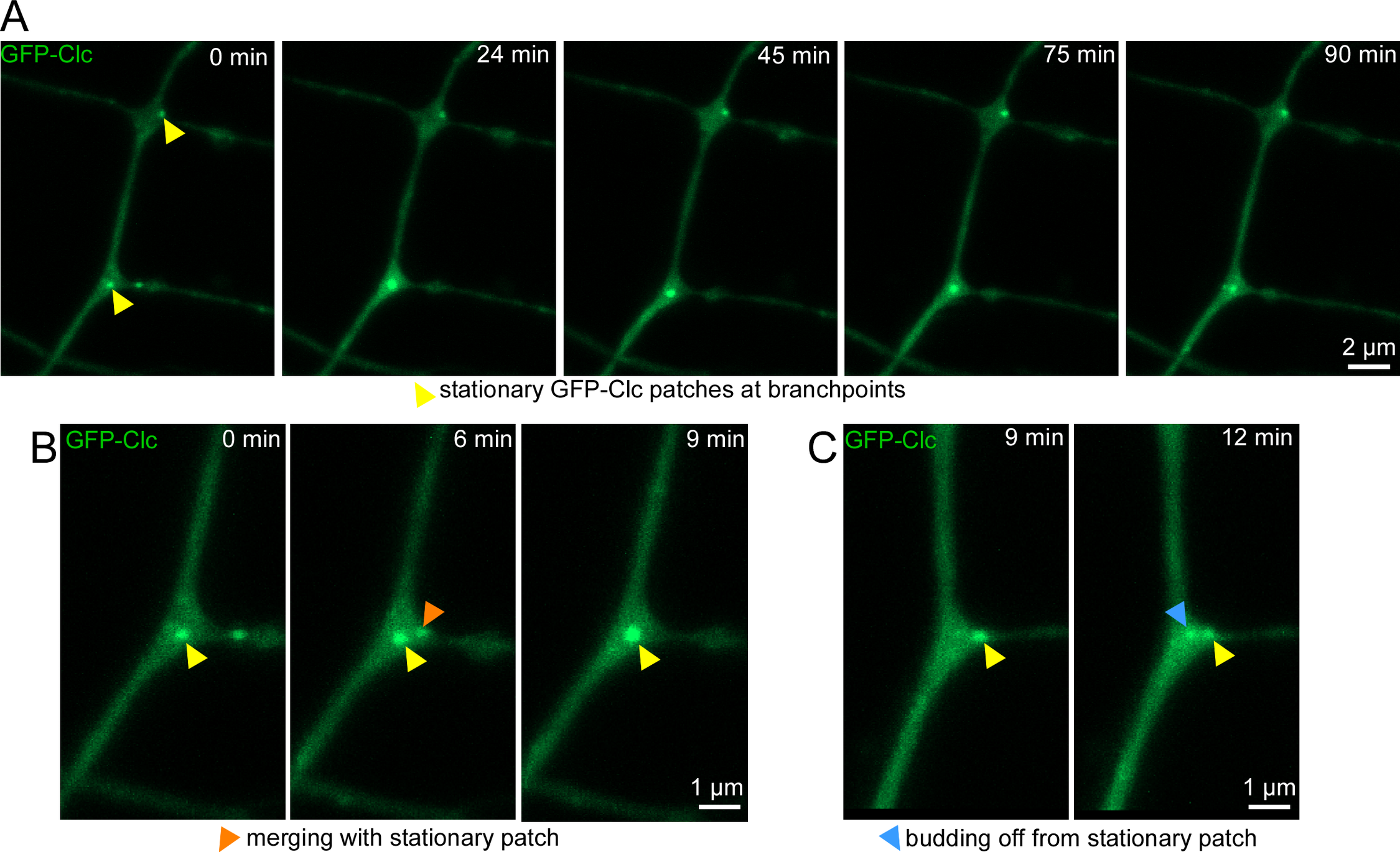

Supplement: S4 Fig — (A) Images at different time points from S4 Movie that show some GFP-Clc puncta remain stationary (yellow arrowheads) at dendrite branch points. The time-lapse movie was acquired from a larva mounted using the LarvaSPA method [64]. (B) Orange arrowhead points to a puncta that merges with the stationary one and makes the stationary one brighter. (C) Blue arrowhead points to a puncta that buds off from the stationary one and makes the stationary one dimmer. (TIF) [file pbio.3002973.s004.tif]

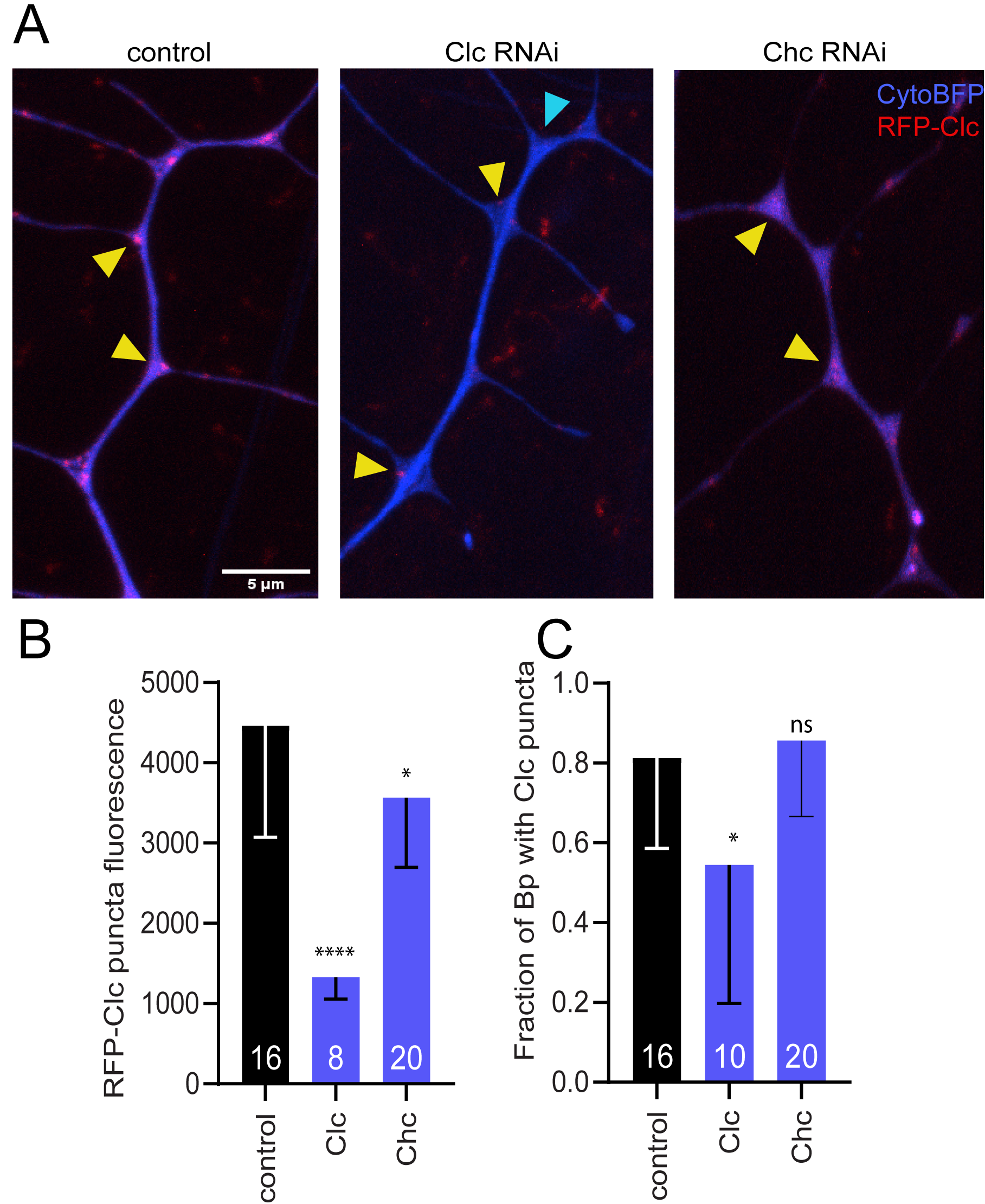

Supplement: S5 Fig — (A) Example images ddaE dorsal dendrites expressing RFP-Clc and cyto-GFP with Tmc-Gal4. Control and clathrin RNA hairpin expression was also driven with 221-Gal4. Yellow arrowheads indicate branch points with RFP-Clc puncta and blue arrowheads indicate branch points without puncta. (B and C) The intensity of RFP-Clc puncta at branch points was measured and occupancy of branch points with a puncta of any intensity above background was counted. Numbers on the graphs are number of cells analyzed and one-way ANOVA was used to compare conditions. Error bars show standard deviation and underlying data is in S2 Table. (TIF) [file pbio.3002973.s005.tif]

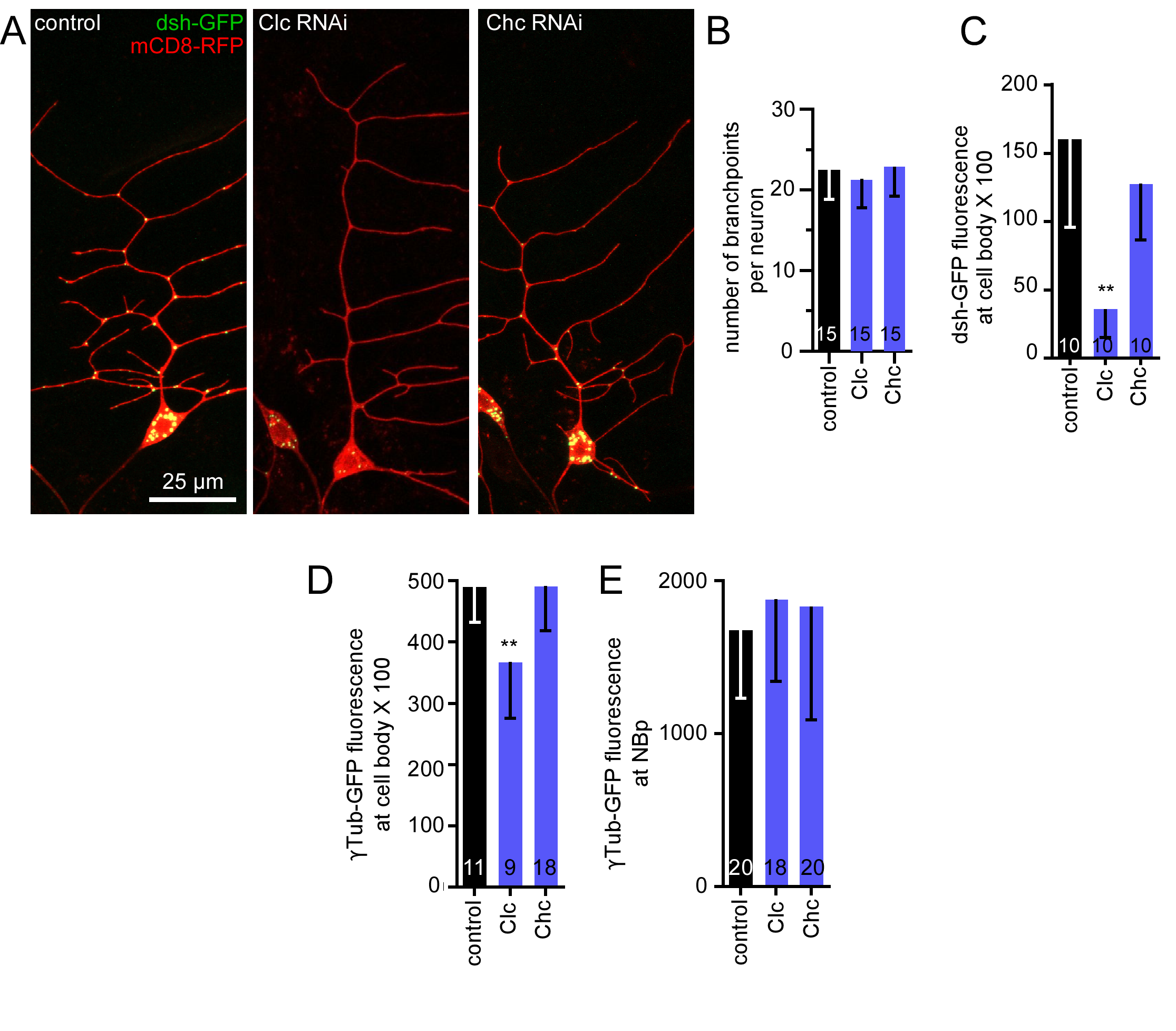

Supplement: S6 Fig — (A) Representative overview images of control, Clc, and Chc-RNAi in ddaE neurons using the 221-Gal4 driver. The neuronal plasma membrane is labeled with UAS-mCD8-RFP and UAS-dsh-GFP puncta can be seen at dendrite branch points. Clc RNAi reduced the cell body fluorescence of dsh-GFP in some cases including the example shown here. (B) Quantitation of the total number of branch points in each neuron. There is no significant difference across the different categories with a Mann–Whitney test. The numbers above the bar graphs represent the number of neurons quantitated in each category. (C) The intensity of dsh-GFP fluorescence was measured in the cell body. (D) Quantitation of γTub-GFP in the cell body in ddaE neurons expressing clathrin RNAi hairpins. Overview images are shown in Fig 8. A Mann–Whitney test was used to compare cell body fluorescence across genotypes. (E) Fluorescence values between branch points for the data set shown in Fig 8B is shown. No change was detected when clathrin levels were reduced; underlying data is contained in S2 Table. (TIF) [file pbio.3002973.s006.tif]
